# Supplementary material for: The behavioral driving mechanism of ecological co-management among multiple subjects from the perspective of social network embedding: Evidence from coffee-producing areas in China
Source: PLoS One. 2026 Mar 2;21(3):e0343504. doi: 10.1371/journal.pone.0343504 (PMC12952628; doi:10.1371/journal.pone.0343504)
Supplement: S1 Appendix — (DOCX) [file pone.0343504.s001.docx]

# Appendix A

**Table A1 Reliability analysis results**

| **Latent variables** | **Observed variables** | **Cronbach's α** |
| --- | --- | --- |
| IBA | IBA1 | 0.912 |
|  | IBA2 |  |
|  | IBA3 |  |
|  | IBA4 |  |
|  | IBA5 |  |
|  | IBA6 |  |
| SN | SN1 | 0.883 |
|  | SN2 |  |
|  | SN3 |  |
|  | SN4 |  |
|  | SN5 |  |
| PBC | PB1 | 0.833 |
|  | PB2 |  |
|  | PB3 |  |
|  | PB4 |  |
| PO | PO1 | 0.970 |
|  | PO2 |  |
|  | PO3 |  |
| ENW | ENW1 | 0.795 |
|  | ENW2 |  |
|  | ENW3 |  |
| SNW | SNW1 | 0.818 |
|  | SNW2 |  |

**TableA2 Rotated component matrix**

| **Variables** | **Component 1** | **Component 2** | **Component 3** | **Component 4** | **Component 5** | **Component 6** | **Communality** |
| --- | --- | --- | --- | --- | --- | --- | --- |
| IBA1 | 0.803 |  |  |  |  |  | 0.757 |
| IBA2 | 0.795 |  |  |  |  |  | 0.770 |
| IBA3 | 0.858 |  |  |  |  |  | 0.763 |
| IBA4 | 0.825 |  |  |  |  |  | 0.704 |
| IBA5 | 0.821 |  |  |  |  |  | 0.746 |
| IBA6 | 0.703 |  |  |  |  |  | 0.651 |
| SN1 |  | 0.811 |  |  |  |  | 0.756 |
| SN2 |  | 0.816 |  |  |  |  | 0.746 |
| SN3 |  | 0.785 |  |  |  |  | 0.659 |
| SN4 |  | 0.839 |  |  |  |  | 0.731 |
| SN5 |  | 0.792 |  |  |  |  | 0.663 |
| PBC1 |  |  | 0.833 |  |  |  | 0.754 |
| PBC2 |  |  | 0.765 |  |  |  | 0.668 |
| PBC3 |  |  | 0.763 |  |  |  | 0.645 |
| PBC4 |  |  | 0.807 |  |  |  | 0.754 |
| PO1 |  |  |  | 0.796 |  |  | 0.925 |
| PO2 |  |  |  | 0.759 |  |  | 0.913 |
| PO3 |  |  |  | 0.785 |  |  | 0.923 |
| ENW1 |  |  |  |  | 0.794 |  | 0.761 |
| ENW2 |  |  |  |  | 0.823 |  | 0.742 |
| ENW3 |  |  |  |  | 0.756 |  | 0.718 |
| SNW1 |  |  |  |  |  | 0.872 | 0.854 |
| SNW2 |  |  |  |  |  | 0.854 | 0.825 |
| Cumulative Variance Contribution Rate | 75.781 | | | | | | |

**TableA3** The content of each dimension in the questionnaire

| **Construct** | **Code** | **Measurement Items** |
| --- | --- | --- |
| Individual Behavioral Attitude (IBA) | IBA1 | Effective ecological co-management is crucial for protecting the coffee plantation's environment. |
|  | IBA2 | Participating in ecological co-management brings significant benefits to the community. |
|  | IBA3 | Active participants in co-management deserve tangible rewards and recognition. |
|  | IBA4 | My participation in ecological co-management can positively influence others' attitudes. |
|  | IBA5 | I am confident in the current level of ecological co-management. |
|  | IBA6 | I have strong confidence in the region's future ecological development. |
| Subjective Norms (SN) | SN1 | My social circle's attitude towards ecological co-management influences my decision. |
|  | SN2 | Ecological co-management initiatives align with the accepted behavioral norms of our society. |
|  | SN3 | My family and friends support my participation in ecological activities. |
|  | SN4 | Compared with other villages, our village has stronger ecological co-management ability. |
|  | SN5 | I feel very good as a member of the ecological management. |
| Perceived Behavioral Control (PBC) | PBC1 | I possess sufficient capability to participate in ecological co-management activities. |
|  | PBC2 | I have adequate time to engage in ecological co-management activities. |
|  | PBC3 | Our village can always handle collective affairs, such as environmental protection. |
|  | PBC4 | In the face of differences of opinion, residents can always solve them well through consultation. |
| Perception of Behavioral Outcome (PO) / Intention | PO1 | Participating in co-management enhances my sense of community identity and responsibility. |
|  | PO2 | I am willing to actively participate in ecological co-management. |
|  | PO3 | I would like to call on my family and friends around me to participate in ecological co-management.. |
| Emotional Network (ENW) | ENW1 | Participating in co-management activities gives me a sense of satisfaction and fulfillment. |
|  | ENW2 | Co-management activities strengthen my emotional connections with other residents. |
|  | ENW3 | I experience positive emotions during the ecological co-management process. |
| Suggestive Network (SNW) | SNW1 | I frequently exchange recommendations about ecological activities with my social network. |
|  | SNW2 | Co-management activities foster closer cooperative relationships within my social network. |
