# Supplementary material for: The behavioral driving mechanism of ecological co-management among multiple subjects from the perspective of social network embedding: Evidence from coffee-producing areas in China
Source: PLoS One. 2026 Mar 2;21(3):e0343504. doi: 10.1371/journal.pone.0343504 (PMC12952628; doi:10.1371/journal.pone.0343504)
Supplement: Attachment — Submitted filename: PLOS ONE review 2025-11-27.pdf [file pone.0343504.s002.pdf]

PLOS ONE REVIEW

PONE-D-25-25310

November 2025

Xiumei Xu

The behavioral driving mechanism of ecological co-management among multiple subjects from the perspective of social network embedding: evidence from coffee-producing areas in China

EXECUTIVE SUMMARY OF STUDY DESIGN

The study aims to provide insight into the behavioral mechanisms explaining why people engage in ecological co-management (as stakeholders), with the hope of shaping policy to encourage such desirable behavior. Specifically, the intention to participate in such activity (PO), treated as a latent variable, is hypothesized to be directly influenced by 5 other latent variables (ENW, SNW, IBA, SN, PBC), conceptually rooted in the theories of planned behavior (TPB) and of social network embeddedness (SNET). Two of these variables (ENW, SNW) are also hypothesized to (positively) influence the other 3 (IBA, SN, PBC), thus exerting also indirect influence on the latent variable of interest (PO). The Authors proceed to test their hypotheses by means of Structural Equation Modelling (SEM), where observed variables, related to the latent variables, are answers to questions disseminated among stakeholders (residents, managers, government officials, NGOs) in Lujiangba coffee plantation base in Yunnan, China.

The dataset consists of 137 (93.8%) participants' valid responses to a questionnaire consisting of 23 questions (incl. 3 questions representing PO), with responses on a Likert scale from 1 to 5 (question IBA2 seems to be an open question, but in the dataset it appears to follow Likert scale). Each question and answer is assigned to exactly one latent variable.

Answers to questions corresponding to the same latent variable show sufficiently high correlation, and to different latent variables – appropriately low correlation. Answers associated with the variable of primary interest, PO, exhibit elevated correlations with answers corresponding to the other latent variables. A participant's answers to

questions related to the same latent variable are presumably averaged to obtain estimate of the value of that latent variable for that participant.

The authors then iteratively estimate the hypothesized model parameters – path coefficients (only those presumed a priori to differ from zero), which determine relationships between the investigated variables – of their (presumably linear) model. With Structural Equation Modelling (SEM), parameters are typically optimized with respect to covariances between all the latent variables, rather than with respect to prediction of a selected target (response, independent) variable from other available (explanatory) variables. The hypothesized model is fitted to all available data at once and is not validated on an out-of-sample test set, as would be more typical of a primarily predictive regression model. Goodness-of-fit of the whole model is not evaluated, significance is only assessed for its individual coefficients, using Bias-Corrected and Accelerated (BCa) bootstrapping to obtain a T statistic for every coefficient.

GENERAL COMMENTS

Overall the manuscript is well-written, with good structure. It is clear that the Authors have dedicated appropriate effort to conduct and present a thorough analysis. Most if not all statistical procedures expected with Structural Equation Modelling have been conducted, though some additional clarification or revision would be desirable.

Considering the topic and domain of application of the study, it is particularly important to keep in mind the wide, interdisciplinary audience of PLOS ONE, potentially unfamiliar with the presented methods, as well as that Structural Equation Modelling (SEM) is not free from controversy, or at least misunderstandings. It involves a degree of arbitrariness in model construction, and combined with idiosyncrasies of questionnaire design, even if well-executed and providing novel insights, it is still advisable to remain conservative with respect to accepting it as conclusive evidence of general principles, especially in context of causality. Deciphering human behavior is an inherently difficult and elusive task, so a (greater) degree of reservedness when drawing conclusions is to be expected, and perfectly acceptable.

The remainder of this review is split in three parts:

A. Main concerns (required revisions)
B. Request for additional details
C. Optional suggestions

A. MAIN CONCERNS

The main concerns in this review pertain to two aspects: assessment of statistical significance in presence of multiple-hypotheses testing, and formulation of conclusions drawn from the data.

A1. Potential overestimation of statistical significance

The Authors tested 17 (sub)hypotheses, each with a separate Student's T-test, each at the 5% significance level, to assess whether each of the 11 estimated model parameters (characterizing the hypothesized direct relationships between latent variables) differ significantly from zero, and whether the 6 additional indirect paths are also significant (it is not entirely clear what specific random variable is being tested in the latter 6 tests; presumably the product of coefficients comprising the tested path). The Authors clearly indicate the p-value and T-statistic for each individual test separately, which allows the Readers to make their own calculations and independent inference at any level of significance, should they be inclined to do so. Nevertheless, when discussing stated conclusions, it is important to consider the level of significance also in context of the whole experiment; especially considering that at least some, if not all, of the hypotheses are related to each other. The experiment-wise Type-I error rate (statistical significance level for the whole model), that is, the probability that at least one of the significant test results is incorrect (at least one of the coefficients confirmed to be significant is actually not) is considerably higher than 5%.

To illustrate the point, let us assume for simplicity, just for a moment, that all 17 tests give a significant result, each with 5% probability that its result is incorrect. Then (assuming the tests are independent; which they may not be, here, but let us set that aside for now) the probability of "an undesirable outcome", undermining the model in any way, i.e. of at least one test giving an incorrectly significant result (indicating a relationship between variables exists and is significant, when in reality it is not), equals 100% – probability of "the desirable outcome" that all tests give a correctly significant result, which is 100% – 95% ^ 17 = 58%. This 58% is the significance level for the whole experiment (this marked increase from 5% is often referred to as 'alpha inflation'). It does not matter how many tests actually come out significant, because the significance threshold may be determined prior to the experiment.

When discussing levels of significance or confidence, many Readers may expect that it is the overall, experiment-wise error rate that is controlled at the level stated in the paper. Again, to their credit, the Authors have not made such misleading claims and have in fact clearly and unambiguously stated all statistics. But with respect to the conclusions drawn from these statistics, to have 95% confidence in the formulation of

the model as a whole, with all its coefficients, requires application of a more conservative threshold than 5% in all the individual tests.

Probably the simplest way to address the issue is to apply the Bonferroni correction to the significance level of every individual test (that is, divide the significance level by the number of conducted tests), and re-evaluate the significance of each hypothesis. Some would still remain significant, but many would not. The Bonferroni correction may be too conservative in this context, so if the Authors wish, they could explore other alternatives (for example, Holm's step-down procedure). But I would be uncomfortable leaving it as it is, without at least explicitly discussing the issue, for the benefit of less statistically-inclined Readers. Especially considering the rather strong statements with respect to drawn conclusions.

A2. Potential overstatement of conclusions (esp. w.r.t. causality)

Causality is notoriously difficult to establish, and in Structural Equation Modelling, it tends to be assumed from theory. Here, for example, it is assumed that SNW and ENW influence IBA, SN and PBC rather than vice versa. The empirical results alone describe only the strength of the association between these variables, but not its direction. Ignoring the theory behind the experiment, one could construct a similar but different model, in which it is the IBA, SN and PBC that influence PO directly or indirectly through SNW and ENW. Such a model would receive exactly the same empirical support with the methods presented in this paper (if the Authors disagree, please elaborate and expand on the methodology; cf. also section B of this review). Therefore, any conclusions about cause and effect in this study are based on theoretical assumptions rather than empirical evidence.

As an example, let us consider the statement from line 381:

"The structural equation modeling results reveal that a statistically significant positive relationship exists between ENW and IBA (t=3.27, p=0.001), demonstrating that emotional networks exert a substantial influence on the formation of positive attitudes toward ecological co-management. "

Only the first part of the sentence reflects the empirical results, while the later part (from “demonstrating that”) reflects the theoretical assumption. The latter part of the statement is not proven or implied by the former part.

Likewise, on line 506:

“The analysis has demonstrated that both types of social networks exert significant positive effects on the key TPB constructs.”

"the affective components captured by the ENW2 measurement item have been demonstrated to455

facilitate the formation of favorable cognitive evaluations regarding co-management behaviors."

On line 533, in turn, there is a **comparison**:

"*both IBA and PBC exhibit stronger effects on stakeholders' participation intentions than SN*"

The strength of the relationship between SN and PO was not compared, statistically, to the strength of the relationship between PBC and PO, or IBA and PO, so this statement should be framed as exploratory analysis or observation to be further investigated in the future, rather than a definitive conclusion. Alternatively, an additional, explicit statistical test, or rather series of tests (Tukey's procedure could perhaps be applicable), may be conducted to establish whether the differences between coefficients are significant – but please bear in mind the earlier remarks about alpha inflation with multiple testing.

As mentioned at the beginning, the nature of the experiment does not, strictly speaking, involve **prediction**, or in particular, evaluation of predictive performance. Therefore the following statements are somewhat controversial at best (esp. the third one, as it implies statistical comparison of predictive power):

- "the analysis indicates that ENW significantly predicts SN" (line 388)
- "ENW significantly predicts SN" (line 388)
- "The analytical outcomes reveal that IBA serves as the most potent predictor of PO ($t=3.654$, $p<0.001$)" (line 422)
- "all three mediating variables were found to be significant predictors of participation intentions" (line 515)

On line 528, we read:

"The results demonstrate that (...) ENW strengthens IBA, SN, and PBC by fostering emotional bonds and collective identity. Meanwhile, SNW improves IBA, SN, and PBC through information dissemination and social influence."

Firstly, again, the results do not demonstrate whether SNW improves IBA, SN and PBC, or whether IBA, SN and PBC improve SNW. Secondly, the experiment investigates whether there is any relationship between SNW and IBA, SN and PBC, it does not

determine the **mechanism** of that relationship. It examines “whether”, not “how”, SNW (or ENW) – according to theory – may influence the other three variables. Arguably, this is the section in which general conclusions are presented, with a degree of flexibility with respect to implications, discussion, etc., but still, it would be desirable to draw a clearer distinction between what directly follows from the empirical analysis, and what constitutes a wider, general interpretation and discussion of potential (not conclusively determined) mechanisms.

A similar example is found on line 402:

“*The analytical results further demonstrate that SNW exerts a significant positive effect on IBA (t=3.132, p=0.002). This relationship can be attributed to the dual mechanisms of information dissemination and social influence operating within suggestive networks, where inputs from community opinion leaders substantially shape participants' cognitive frameworks.*”

In this case (aside from implied causality), arguably, the phrasing “can be attributed to” seems to indicate an interpretation, but a more explicit distinction would be preferable.

In general, it is good that the Authors discuss possible mechanisms. However, it is also desirable to **separate empirical findings from their interpretation and discussion** more clearly, so that even Readers who do not read the paper in full still have appropriate understanding of what constitutes the empirical findings, and what constitutes their theoretical context. This doesn’t have to be a spatial separation, just slightly different wording.

On another note – a challenge with drawing conclusions in this and similar studies lies also with the definition (in particular, scope) of latent variables, and how much of their interpretation is actually captured by the observed variables. Latent variables are abstract concepts, which are only imperfectly expressed through the observed variables, and in the interpretation of results, one must bear that reservation in mind. The interpretation of results cannot rely on a wider understanding of the latent concept that the specific observed variables allow.

B. REQUEST FOR ADDITIONAL DETAILS

Future researchers may wish to reproduce the same results, on the same dataset, to confirm the agreement in methodology, before they apply a modification to the methods, investigate additional hypotheses, or replicate the study in another country, or countries, to investigate regional differences in the examined aspects. Therefore adding some technical details and clarifications would be highly desirable, and also required, in accordance with PLOS Publication Criterion no. 3: "Methods and reagents must be described in sufficient detail for another researcher to reproduce the experiments described." Below is a list of items that would ideally be added to the main text or to the appendix, or otherwise (if not feasible) explained here.

1. **Mathematical formulas** and other details allowing full replication.
   They do not have to be very formal, but they need to describe all procedures completely. Preferably to a Reader unfamiliar with Structural Equation Modelling, or one not in possession of the cited software. There are, for example, multiple ways to approach the problem of coefficient estimation, specific random seeds may be used in bootstrapping, etc., so obtaining the exact same coefficient values as the Authors did is not currently possible.
   Selected specific questions (not an exhaustive list):
   a. What is the exact objective function being optimized in process of estimation of the coefficients of the model?
   b. What is the nature of the estimated coefficients? Presumably the model is represented by a set of linear equations? It would be good to have them written out explicitly, in addition to the flowchart with arrows.
   c. How exactly is the significance of the indirect paths assessed?
      How is the T-statistic calculated for the indirect paths, what is the (|O/STDEV|) in Table 4?
2. (optional) It would be desirable to add the **intermediate dataset** to the raw data uploaded online, that is, the outcome of the iterative model estimation, with the numbers used to calculate the T statistics. In other words, everything required to follow all calculations exactly, from original raw data to final estimates.
3. **Original questions** (in original spelling, in the language in which they were asked, e.g. if there was a typo, it should be retained exactly as it was) should be included in the dataset or the appendix, just like the demographic questions are, in case of some future doubts about translation.
4. What is the (Likert) **scale of the answers**, how was it formulated? Does 1 mean Strongly Agree and 5 Strongly Disagree, in every question? Was there any transformation, truncation, any modification to originally recorded values at all? Were any values missing, if so, how many, which, how were they handled?
5. How are answers to individual questions in the questionnaire averaged to obtain values of the **latent variables**? Arithmetic mean, median?

Optional additions:

6. It would be very desirable (but not absolutely compulsory) to add another column to the online dataset, specifying whether the answers from a participant were obtained **online or in-person**. Ideally, a code designated the interviewer would be added (so that one could distinguish which participants were interviewed by the same person).
7. If possible, another column could be added, reporting **relationships between participants**: was the participant interviewed together with another participant (if so, which)? Have multiple members of the same family, or housemates, been invited to the survey? It is conceivable that people who live together, or are close friends, partners, co-workers, etc., share certain attitudes and convictions, or that they fill out the questionnaire together, in which case their answers may be influenced by the concern about the opinion of the other participant about their answers. Such additional information could therefore provide valuable insights in the future, plus, would allow to assess the independence of the sampled data, even if the information is only available for a subset of participants. But I understand that participants may not wish to provide such information, there may be additional personal data protection ramifications, etc., so please consider it more a suggestion that request.
8. Was the survey anonymous, were any participants personally known to the interviewers?
9. Were any participants (if so, which) already actively involved in the co-management? Again, this is optional.

C. OPTIONAL SUGGESTIONS AND COMMENTS

Overall, the text is well-written; even if it could benefit from a little less jargon in some places, especially the Introduction.

It lack a discussion of limitations of methodology, and statistical analysis in particular.

A short paragraph on future work (what else could be done on this subject, logical next research questions to investigate, alternative approaches or models to pursue, perhaps with other data or resources) would help put the study into perspective.

The ending is slightly abrupt. A distinctive, impactful **closing paragraph** referring back to the main objectives and accomplishments would improve overall impression.

The legends of the demographic data pie charts (**Figure 2**) are not entirely legible, due to the use of similar colors and no alternative way to connect the labels with the parts of the chart, other than by reading the main text. Perhaps adding a background line pattern would be helpful, or positioning labels next to the pieces that they correspond to. Such improved accessibility may be particularly appreciated by color-blind (or visually-impaired in other ways) Readers.

I found **Figure 3** (flowchart illustration of the model) slightly confusing at first; it cleared up after understanding the model from reading the text. I understand it is difficult to graphically distinguish Hypothesis 2 from Hypothesis 4. So this is just an optional suggestion: I would consider repositioning the boxes so that all arrows point from left to right. That would focus the Reader's attention immediately on the meaning rather than direction of the arrows. It would also make the absence of cycles immediately clear.

Whatever the Authors' decision with respect to Figure 3, I would strongly advise to keep **Figure 4** consistent with Fig. 3, that is, for it to have the same layout of latent variables. Again, I would personally prefer to see the ENW and SNW on the left and IBA, SN, PBC in the middle, but I am not particular about this.

The **order of latent variables** should be the same in every table and figure. For example, in Table 2, it differs for no apparent reason.

Table 3 could also have a slightly different order of rows, for example, corresponding to the order of paths in Fig. 4, starting with top left. But if there is another pattern I did not notice at first, that is fine too.

Typos:

- Line 514: “Furthermore, Consistent” – capitalization error
- Line 384: SNWT instead of SNET (I presume)
- Online dataset: IBA questions labelled as IA
