# Supplementary material for: The behavioral driving mechanism of ecological co-management among multiple subjects from the perspective of social network embedding: Evidence from coffee-producing areas in China
Source: PLoS One. 2026 Mar 2;21(3):e0343504. doi: 10.1371/journal.pone.0343504 (PMC12952628; doi:10.1371/journal.pone.0343504)
Supplement: Attachment — Submitted filename: Response document.docx [file pone.0343504.s003.docx]

**Response document**

**Manuscript Number:** PONE-D-25-25310
**Title:** The behavioral driving mechanism of ecological co-management among multiple subjects from the perspective of social network embedding: evidence from coffee-producing areas in China

**Journal:** PLOS ONE

| **Response to the editor** | |
| --- | --- |
| **Editor’s comments** | **Authors’ response** |
| Dear Dr. XU,  Thank you for submitting your manuscript to PLOS ONE. After careful consideration, we feel that it has merit but does not fully meet PLOS ONE’s publication criteria as it currently stands. Therefore, we invite you to submit a revised version of the manuscript that addresses the points raised during the review process.  Please submit your revised manuscript by Jan 17 2026 11:59PM. If you will need more time than this to complete your revisions, please reply to this message or contact the journal office at [plosone@plos.org](mailto:plosone@plos.org). When you're ready to submit your revision, log on to [https://www.editorialmanager.com/pone/](https://www.editorialmanager.com/pone/) and select the 'Submissions Needing Revision' folder to locate your manuscript file.  Please include the following items when submitting your revised manuscript:   - A rebuttal letter that responds to each point raised by the academic editor and reviewer(s). You should upload this letter as a separate file labeled 'Response to Reviewers'. - A marked-up copy of your manuscript that highlights changes made to the original version. You should upload this as a separate file labeled 'Revised Manuscript with Track Changes'. - An unmarked version of your revised paper without tracked changes. You should upload this as a separate file labeled 'Manuscript'.   If you would like to make changes to your financial disclosure, please include your updated statement in your cover letter. Guidelines for resubmitting your figure files are available below the reviewer comments at the end of this letter.  If applicable, we recommend that you deposit your laboratory protocols in protocols.io to enhance the reproducibility of your results. Protocols.io assigns your protocol its own identifier (DOI) so that it can be cited independently in the future. For instructions see: [https://journals.plos.org/plosone/s/submission-guidelines#loc-laboratory-protocols](https://track.editorialmanager.com/CL0/https://journals.plos.org/plosone/s/submission-guidelines%23loc-laboratory-protocols/1/010f019b1061a57d-34cb25bf-49f6-4b63-b31a-24345018d592-000000/c9uuFO_ChXFK-zVOnuEExzn-A5qlpf-ASK9BFUB5VXw=241).  Additionally, PLOS ONE offers an option for publishing peer-reviewed Lab Protocol articles, which describe protocols hosted on protocols.io. Read more information on sharing protocols at [https://plos.org/protocols?utm_medium=editorial-email&utm_source=authorletters&utm_campaign=protocols](https://track.editorialmanager.com/CL0/https://plos.org/protocols?utm_medium=editorial-email&utm_source=authorletters&utm_campaign=protocols/1/010f019b1061a57d-34cb25bf-49f6-4b63-b31a-24345018d592-000000/jgY6Ybew5puYFzpgnXtwAUYuD4jxsqzfg6yCEfN2FLA=241).  We look forward to receiving your revised manuscript.    Kind regards,  Umberto Baresi, Ph.D. Academic Editor PLOS ONE  Journal Requirements:  When submitting your revision, we need you to address these additional requirements.  1. Please ensure that your manuscript meets PLOS ONE's style requirements, including those for file naming. The PLOS ONE style templates can be found at  [https://journals.plos.org/plosone/s/file?id=wjVg/PLOSOne_formatting_sample_main_body.pdf](https://journals.plos.org/plosone/s/file?id=wjVg/PLOSOne_formatting_sample_main_body.pdf) and  [https://journals.plos.org/plosone/s/file?id=ba62/PLOSOne_formatting_sample_title_authors_affiliations.pdf](https://journals.plos.org/plosone/s/file?id=ba62/PLOSOne_formatting_sample_title_authors_affiliations.pdf)  2. Please provide additional details regarding participant consent. In the ethics statement in the Methods and online submission information, please ensure that you have specified (1) whether consent was informed and (2) what type you obtained (for instance, written or verbal, and if verbal, how it was documented and witnessed). If your study included minors, state whether you obtained consent from parents or guardians. If the need for consent was waived by the ethics committee, please include this information.  If you are reporting a retrospective study of medical records or archived samples, please ensure that you have discussed whether all data were fully anonymized before you accessed them and/or whether the IRB or ethics committee waived the requirement for informed consent. If patients provided informed written consent to have data from their medical records used in research, please include this information.  3. Thank you for stating the following in the Acknowledgments Section of your manuscript:  “This work is supported by the Yunnan Province Philosophy and Social Science Planning (No. ZX2024ZD10).”  We note that you have provided funding information that is not currently declared in your Funding Statement. However, funding information should not appear in the Acknowledgments section or other areas of your manuscript. We will only publish funding information present in the Funding Statement section of the online submission form.  Please remove any funding-related text from the manuscript and let us know how you would like to update your Funding Statement. Currently, your Funding Statement reads as follows:  “This work is supported by the Yunnan Province Philosophy and Social Science Planning (No. ZX2024ZD10).”  Please include your amended statements within your cover letter; we will change the online submission form on your behalf.  4. Thank you for stating the following financial disclosure:  “This work is supported by the Yunnan Province Philosophy and Social Science Planning (No. ZX2024ZD10).”  Please state what role the funders took in the study.  If the funders had no role, please state: "The funders had no role in study design, data collection and analysis, decision to publish, or preparation of the manuscript."  If this statement is not correct you must amend it as needed.  Please include this amended Role of Funder statement in your cover letter; we will change the online submission form on your behalf.  5. We note that Figure 1 in your submission contain map images which may be copyrighted. All PLOS content is published under the Creative Commons Attribution License (CC BY 4.0), which means that the manuscript, images, and Supporting Information files will be freely available online, and any third party is permitted to access, download, copy, distribute, and use these materials in any way, even commercially, with proper attribution. For these reasons, we cannot publish previously copyrighted maps or satellite images created using proprietary data, such as Google software (Google Maps, Street View, and Earth). For more information, see our copyright guidelines: [http://journals.plos.org/plosone/s/licenses-and-copyright.](http://journals.plos.org/plosone/s/licenses-and-copyright.)  We require you to either (1) present written permission from the copyright holder to publish these figures specifically under the CC BY 4.0 license, or (2) remove the figures from your submission:  1. You may seek permission from the original copyright holder of Figure(s) [#] to publish the content specifically under the CC BY 4.0 license.  We recommend that you contact the original copyright holder with the Content Permission Form ([http://journals.plos.org/plosone/s/file?id=7c09/content-permission-form.pdf](http://journals.plos.org/plosone/s/file?id=7c09/content-permission-form.pdf)) and the following text:  “I request permission for the open-access journal PLOS ONE to publish XXX under the Creative Commons Attribution License (CCAL) CC BY 4.0 ([http://creativecommons.org/licenses/by/4.0/](http://creativecommons.org/licenses/by/4.0/)). Please be aware that this license allows unrestricted use and distribution, even commercially, by third parties. Please reply and provide explicit written permission to publish XXX under a CC BY license and complete the attached form.”  Please upload the completed Content Permission Form or other proof of granted permissions as an "Other" file with your submission.  In the figure caption of the copyrighted figure, please include the following text: “Reprinted from [ref] under a CC BY license, with permission from [name of publisher], original copyright [original copyright year].”  2. If you are unable to obtain permission from the original copyright holder to publish these figures under the CC BY 4.0 license or if the copyright holder’s requirements are incompatible with the CC BY 4.0 license, please either i) remove the figure or ii) supply a replacement figure that complies with the CC BY 4.0 license. Please check copyright information on all replacement figures and update the figure caption with source information. If applicable, please specify in the figure caption text when a figure is similar but not identical to the original image and is therefore for illustrative purposes only.  The following resources for replacing copyrighted map figures may be helpful:  USGS National Map Viewer (public domain): [http://viewer.nationalmap.gov/viewer/](http://viewer.nationalmap.gov/viewer/)  The Gateway to Astronaut Photography of Earth (public domain): [http://eol.jsc.nasa.gov/sseop/clickmap/](http://eol.jsc.nasa.gov/sseop/clickmap/)  Maps at the CIA (public domain): [https://www.cia.gov/library/publications/the-world-factbook/index.html](https://www.cia.gov/library/publications/the-world-factbook/index.html) and [https://www.cia.gov/library/publications/cia-maps-publications/index.html](https://www.cia.gov/library/publications/cia-maps-publications/index.html)  NASA Earth Observatory (public domain): [http://earthobservatory.nasa.gov/](http://earthobservatory.nasa.gov/)  Landsat: [http://landsat.visibleearth.nasa.gov/](http://landsat.visibleearth.nasa.gov/) USGS EROS (Earth Resources Observatory and Science (EROS) Center) (public domain): [http://eros.usgs.gov/#](http://eros.usgs.gov/) Natural Earth (public domain): [http://www.naturalearthdata.com/](http://www.naturalearthdata.com/)  6. If the reviewer comments include a recommendation to cite specific previously published works, please review and evaluate these publications to determine whether they are relevant and should be cited. There is no requirement to cite these works unless the editor has indicated otherwise. | Dear Prof. Umberto Baresi,  Thank you for your email and for giving us the opportunity to revise our manuscript for possible publication in PLOS ONE. We sincerely appreciate the detailed feedback from the editors and reviewers, which has been instrumental in improving the quality and clarity of our work.  We have carefully addressed all the points raised in the editorial and reviewer comments, as well as the journal requirements. The key revisions include:   1. Comprehensive restructuring of the theoretical framework and literature review to strengthen conceptual clarity. 2. Significant refinement of the conceptual model and methodological description to enhance transparency and rigor. 3. Full compliance with PLOS ONE style and formatting guidelines. 4. Provision of detailed ethical approval and participant consent information. 5. Correction of funding information placement and inclusion of the required funder role statement. 6. Removal of Figure 1 to comply with copyright guidelines, with the relevant information concisely described in the text.   All changes have been meticulously implemented and are clearly indicated in the tracked-changes version of the manuscript. A point-by-point response to each comment, detailing our specific revisions and their locations in the text, is provided in the separate "Response to Reviewers" document.  We appreciate the valuable feedback that has guided us in enhancing the quality of our work. We look forward to your further suggestions and hope for a favorable consideration of our revised submission. Thank you once again for the opportunity to improve our manuscript.  Best wishes,  Yours sincerely,  Xiumei XU, Ph.D. |

| **Response to the Editor** | |
| --- | --- |
| **Editor’s comments** | **Authors’ response** |
| Dear Authors,  I would like to thank you for submitting your work for publication in PLOS ONE.  After this first round of reviews, I am pleased to convey that your work has been deemed as suitable for publication. The anonymous reviewers indicated that for your work to meet its full potential, it requires major restructuring in theoretical framing, literature review, conceptual model design, and methodological transparency.  Specifically, "the conceptual foundations and operationalization require substantial revision".  Please consider also the attached document, in which additional comments from Reviewer #2 are provided.  Please consider all attached feedback before submitting the edited version of your work.  As a requirement for resubmission, please attach the edited version of the manuscript with Changes Tracked in a different color, and a table in which each comment in listed in rows, for which I would ask you to indicate how and where in the document the edits took place.  Thank you. | Dear Editor,  We sincerely appreciate your valuable comments on our manuscript and are pleased to be informed that the manuscript is deemed to basically meet the publication requirements after the first round of review. We have carefully read and thoroughly reflected on all the suggestions put forward by the reviewers, particularly those regarding the need for significant adjustments to the theoretical framework, literature review, conceptual model design, and methodological transparency, as well as the requirement for substantial revisions to the conceptual basis and operationalization.  In accordance with your and the reviewers' recommendations, we have conducted a systematic and comprehensive revision of the manuscript. The key revisions are as follows:  1.Reorganized the theoretical framework to strengthen the integration logic of the Theory of Planned Behavior (TPB) and Social Network Embeddedness Theory (SNET);  2. Reconstructed the literature review section to highlight the evolution of relevant research and the positioning of this study;  3. Optimized the conceptual model and clearly explained the operational definitions and measurement methods of each construct;  4. Supplemented methodological details to enhance the transparency and reproducibility of the analysis process.  5. All revisions have been marked in different colors to clearly show the content and location of modifications;  6. Detailedly listing each reviewer's comment, along with explanations of how we addressed them in the manuscript and the specific locations.  We appreciate the time and professional advice you and the reviewers have invested in improving the quality of this manuscript. We believe that this revision has significantly enhanced the theoretical rigor, structural clarity, and methodological completeness of the paper, making it more consistent with the publication standards of your journal. We are happy to make further improvements if additional suggestions are provided.  Best wishes,  Yours sincerely,  Xiumei XU, Ph.D. |

| **Response to Reviewer 1** | |
| --- | --- |
| **Reviewer 1’s comments** | **Authors’ response** |
| Thank you for the opportunity to review this manuscript. The topic of ecological co-management in agricultural regions is important, and integrating TPB with social network embeddedness has potential value. However, several substantial issues need to be addressed before the manuscript can be considered further. Below I present key concerns, each followed immediately by a recommendation to improve clarity, theoretical rigor, and methodological soundness. | We sincerely thank the reviewer for their insightful and constructive comments, as well as their recognition of the importance and potential of our research topic. We have carefully considered each point raised and have made comprehensive revisions to the manuscript accordingly. Below we provide a point-by-point response to the specific concerns and detail the changes implemented to address them. We believe these revisions have significantly enhanced the clarity, theoretical rigor, and methodological soundness of our work.  Thank you again for your meticulous review—your input has greatly elevated the quality of our work.  Below, we present our point-by-point responses to your comments, detailing the specific modifications made to address each issue. |
| 1. Introduction and Problem Definition  The introduction outlines global ecological issues but does not clearly explain the specific ecological challenges in the Lujiangba coffee region or why ecological co-management is necessary. This limits the contextual relevance of the study.   Recommendation: Strengthen the introduction by describing concrete local ecological problems (e.g., soil erosion, chemical overuse, biodiversity decline) and clarifying why this region is an appropriate case. | Thank you very much for your valuable comments on our manuscript. We highly appreciate your insightful suggestion regarding strengthening the introduction by describing concrete local ecological problems in the Lujiangba coffee region and clarifying the appropriateness of this case. We have carefully revised the introduction section in response to your comments, and the details of the revisions are as follows:  In the original introduction, we only briefly mentioned that the Lujiangba coffee plantation base is a salient case study for ecological governance but failed to elaborate on the specific local ecological challenges and the necessity of ecological co-management. To address this limitation and enhance the contextual relevance of the study, we have added a detailed paragraph that specifies the prominent local ecological problems resulting from long-term intensive coffee cultivation in the Lujiangba area of Gaoligong Mountain, Yunnan Province. Specifically, we have clarified three core ecological challenges:  1. Slope topography and soil erosion during coffee cultivation have led to declining soil fertility and degradation of arable land quality;  2. The heavy reliance on traditional washing methods for coffee cherry processing, coupled with the lack of advanced wastewater treatment facilities, has exerted considerable pressure on the local ecosystem;  3. The monoculture structure of coffee cultivation has significantly reduced local biodiversity, weakening ecosystem stability and resilience.  Additionally, we have supplemented that the waste generated during coffee cherry processing belongs to non-point source pollutants, emphasizing that pollution from coffee cultivation and processing cannot be overlooked. We have also highlighted the close link between ecological governance and the livelihoods of local farmers, which further illustrates the urgent necessity of collaborative ecological management in this region.  The newly added content is as follows.  The coffee cultivation base in the Lujiangba area of Gaoligong Mountain, Yunnan Province, serves as a typical case study for ecological governance. As a representative mountainous coffee-producing region, this area faces prominent local ecological challenges resulting from long-term intensive cultivation. First, slope topography and soil erosion during coffee cultivation have led to declining soil fertility and degradation of arable land quality. Second, the processing of coffee cherries remains heavily reliant on traditional washing methods. Due to the absence of advanced wastewater treatment facilities, this practice exerts considerable pressure on the local ecosystem. Third, the monoculture structure of coffee cultivation has significantly reduced local biodiversity, weakening ecosystem stability and resilience. Additionally, waste generated during coffee cherry processing should also be classified as a non-point source pollutant. Consequently, pollution arising from coffee cultivation and processing cannot be overlooked. Ecological governance is closely linked to the livelihoods of local farmers, making collaborative ecological management an urgent necessity. The effectiveness of governance in this region directly determines the sustainability of the local ecosystem and community well-being. (Page 3; Lines 71-85. Clean version).  These revisions explicitly clarify why the Lujiangba coffee region is an appropriate case for studying multi-stakeholder ecological co-management, as its ecological problems are typical of mountainous coffee-producing areas and closely related to local sustainable development.  We believe that the revised introduction effectively addresses the issue you raised, strengthens the contextual relevance of the study, and lays a more solid foundation for the subsequent research content. Once again, we sincerely appreciate your constructive comments, which have greatly improved the quality of our manuscript. |
| 2. Literature Review and Research Gap  The literature review summarizes TPB and SNET but does not develop a coherent argument leading to a clear research gap. The integration of the two theories is not justified, and existing studies combining networks with behavioral intention are not adequately discussed.   Recommendation: Reorganize the review to (a) describe previous studies on TPB in environmental participation, (b) show how relational embeddedness influences cooperation, and (c) highlight the gap this study addresses. | Thank you sincerely for your insightful comments on the literature review and research gap section of our manuscript. We fully acknowledge the limitations you identified, including the lack of a coherent argument leading to a clear research gap, insufficient justification for integrating TPB and SNET, and inadequate discussion of studies combining networks with behavioral intention. We have thoroughly revised this section in strict accordance with your recommendations, and the detailed revisions are outlined below: First, we have completely reorganized the literature review to form a logical and sequential argument, aligning with your suggestions to (a) describe previous studies on TPB in environmental participation, (b) show how relational embeddedness influences cooperation, and (c) highlight the research gap addressed by this study. Compared with the original review, which separately summarized TPB, SNET, and multi-stakeholder ecological collaborative management without strong logical connections, the revised version adopts a more structured framework with four thematic sections, each building on the previous one to progressively lead to the research gap. Regarding (a) previous studies on TPB in environmental participation, we have expanded this part to systematically elaborate on the core constructs of TPB (attitude, subjective norm, perceived behavioral control) and its extensive applications in various pro-environmental behavior contexts, such as waste sorting, payment for ecosystem services, and environmental governance participation. We have supplemented specific empirical examples to demonstrate TPB's explanatory power. More importantly, we have explicitly pointed out the critical limitation of existing TPB research in the environmental domain—its predominant focus on unidimensional, individual-level analyses, which fails to capture the complex interdependencies in multi-stakeholder collaborative systems and overlooks the influence of social context on individual decision-making. For (b) how relational embeddedness influences cooperation, we have added a dedicated section to clarify the connotation of relational embeddedness (emphasizing trust, reciprocity, and shared norms) and distinguish it from structural embeddedness. We have comprehensively reviewed empirical studies across different fields (including environmental management, venture capital, and industry-university cooperation) to illustrate the positive link between relational embeddedness and cooperative behavior. We have also summarized the core mechanisms through which relational embeddedness fosters cooperation, namely building trust and reducing transaction costs, facilitating information and influence flow, and enforcing norms of cooperation. This section effectively connects the individual-level decision-making logic of TPB with the social context perspective of SNET, laying a foundation for justifying their integration. Third, we have added a new section titled "Theoretical and Empirical Rationale for an Integrated TPB-SNET Framework" to address the insufficient justification for theory integration in the original version. We have reviewed and discussed existing studies that combine social network variables with TPB, such as research on rural residents' engagement in living environment upgrades and participation in old neighborhood regeneration. These studies validate the feasibility of integrating the two theories. Furthermore, we have explicitly justified the integration from three aspects: theoretical complementarity (TPB explains micro-level individual psychological processes, while SNET provides meso-level social structure insights), empirical support (integrated models show improved fit and explanatory power), and direct alignment with addressing the identified research gap. A summary table is also included to clarify the core tenets, contributions, and limitations of TPB and SNET, highlighting the synergistic value of their integration. Finally, regarding (c) highlighting the research gap, the revised section clearly delineates the critical knowledge gap based on the preceding literature review: despite the established link between relational embeddedness and cooperation, the specific mechanisms through which relational embeddedness influences pro-environmental behavioral intentions—particularly the precise pathways through which network trust and ties shape TPB's core constructs (attitude, subjective norm, perceived behavioral control)—remain under-examined in the context of complex ecological governance. We have further clarified how this study addresses this gap by developing and testing a novel integrated TPB-SNET framework that investigates the specific mediating pathways of relational embeddedness, rather than merely adding network variables to a TPB model. In addition, we have supplemented relevant empirical studies and citations throughout the revised section to enhance the academic rigor of the review. The revised literature review now forms a coherent argument: existing TPB research lacks consideration of social context; relational embeddedness is a key driver of cooperative behavior in social contexts; integrating TPB and SNET can complement each other's limitations; and the specific mechanism of relational embeddedness influencing pro-environmental behavioral intentions via TPB constructs is the critical gap this study fills. The revised Section 2 is presented as follows (Pages 5-9; Lines 124-228. Clean version). 2.Literature Review 2.1 Application of the TPB in environmental participation The TPB posits that behavioral intention is the most proximal determinant of actual behavior, and this intention is formed by three core constructs: attitude toward the behavior, subjective norm, and perceived behavioral control [13]. Attitude refers to the degree to which a person has a favorable or unfavorable evaluation of the behavior in question [14]. Subjective norm represents the social pressure an individual perceives from important others (e.g., family, friends, colleagues) to perform or not to perform the behavior [15]. Perceived behavioral control is the individual's perception of their own ability to perform the behavior, which is influenced by factors like resources, opportunities, and skills [16]. TPB has been extensively and fruitfully applied to model and predict a wide range of pro-environmental behaviors. Research across diverse contexts, including waste sorting [17], conservation agriculture [18] and participation in environmental governance [19], consistently demonstrates its robust explanatory power. For instance, a study on rural villagers' willingness to participate in water environment governance in Fujian, China, found that environmental awareness and attitude had a significantly greater impact on participation willingness[20]. Similarly, research on citizens' willingness to pay for urban forest ecosystem services showed that variables such as perceived trustworthiness in the payment mechanism and environmental knowledge, when integrated into the TPB framework, significantly enhanced the model's explanatory power for individuals' willingness to pay [21]. However, a critical limitation of much of the existing TPB literature in the environmental domain is its predominant focus on unidimensional, individual-level analyses. While this approach is effective for understanding isolated behaviors, it often fails to capture the complex dynamics at play within multi-stakeholder collaborative systems, such as those common in ecological governance. This oversight represents a key theoretical gap, as it overlooks how the social context and interpersonal relationships shape an individual's decision to participate. 2.2 The role of SNET in fostering cooperation While TPB provides a robust model for individual decision-making, SNET perspective—specifically the concept of relational embeddedness—offers crucial insights into the social context that facilitates or hinders cooperation [22, 23]. Relational embeddedness refers to the nature and quality of relationships among actors in a network, emphasizing the importance of trust, reciprocity, and shared norms [8]. It is distinct from structural embeddedness, which focuses on an actor's position within a network (e.g., centrality, bridges) [24]. Empirical evidence strongly supports the link between strong, trusting relationships and cooperation. For example, in the healthcare sector, relational embeddedness has been empirically validated as a key antecedent to effective collaboration and organizational learning [25]. Within ecological governance research, SNET has been productively applied to explicate phenomena related to community-based environmental management and collaborative governance arrangements [26]. For instance, empirical investigations have revealed that the density of trust networks and strength of social connections among community members constitute significant predictors of ecological participation motivation [27]. The core mechanisms through which SNET promotes cooperation involve building trust to reduce transaction costs by mitigating risks and uncertainties associated with cooperation, thereby diminishing reliance on formal contracts and monitoring; facilitating the efficient flow of information, resources and influence among embedded actors, which aligns efforts and fosters collective action; and enforcing cooperative norms through networks founded on trust and reciprocity, which generate social expectations that encourage collaboration and deter free-riding. In summary, while the TPB explains the formation of individual behavioral intention, the concept of relational embeddedness from SNET elucidates how the social environment supplies the essential mechanisms, grounded in trust, information flow and normative enforcement, for translating such intention into sustained collective and cooperative action, particularly within complex governance contexts such as ecological management. 2.3 Theoretical and empirical rationale for an integrated TPB-SNET framework The limitations of TPB and SNET when applied in isolation create a compelling rationale for their integration. A review of the literature reveals a growing number of studies that implicitly or explicitly combine social network concepts with behavioral models, providing a foundation for the current study's integrated framework. Several studies have successfully incorporated social network variables into extended TPB models. For instance, research on farmers' technology adoption has demonstrated that incorporating social network structures and perceived risks into an extended TPB model significantly enhances its explanatory power [28]. Another demonstrated that adding the construct of social network embeddedness to the TPB framework effectively predicts residents' participation intentions [27]. These studies validate the argument that social context variables are not merely external influences but are internalized through the core components of the TPB model. The integration of the TPB and SNET is well-justified on both theoretical and empirical grounds [29]. The theoretical complementarity is evident, as TPB provides a micro-level mechanism by focusing on individual cognition and intention, while SNET contributes a meso-level perspective by emphasizing the social structures of relational embeddedness that enable and sustain cooperation [28]. Empirical support further strengthens this integration, as studies incorporating social network variables into TPB frameworks have demonstrated improved model fit and explanatory power compared to models based on either theory alone. This approach directly addresses a key research gap, namely, the limitations of unidimensional models in capturing the complexities of multi-stakeholder collaboration. By explicitly modeling how relational embeddedness shapes the core antecedents of behavioral intention, the integrated framework offers a more nuanced and robust explanation of participation dynamics in contexts such as ecological governance. Table 1 summarizes the core tenets and contributions of the two theories, highlighting the synergistic potential of their integration. Table 1. Comparative analysis of the TPB and SNET **Theory** \| **Core concepts** \| **Primary contribution** \| **Limitations**; **TPB** \| Attitude, Subjective Norm, Perceived Behavioral Control, Intention \| Explains individual-level psychological processes leading to behavior [30]. \| Fails to account for the influence of social structure and relational context on individual decisions.; **SNET** \| Relational Embeddedness, Structural Embeddedness, Trust, Network Ties \| Explains how social context and relationships facilitate or constrain cooperation [31]. \| Provides less insight into the specific cognitive and motivational processes of individual actors. 2.4 Research gaps and contributions of this study Notwithstanding the theoretical utility of both TPB and SNET, a critical knowledge gap persists concerning the specific mechanisms through which relational embeddedness influences pro-environmental behavioral intentions [32]. While it is well-established that relationships matter [33], the precise pathways through which network trust and ties shape attitude, subjective norm and perceived behavioral control remain under-examined, particularly in the context of complex ecological governance. In response to these theoretical constraints, a significant contribution is made by this study through the development and testing of a novel integrated TPB-SNET analytical framework. This model advances beyond the mere inclusion of network variables within a TPB structure by positing and empirically examining specific mediating and moderating pathways through which relational embeddedness operates. These mechanisms are investigated to yield a more detailed and nuanced understanding of stakeholder participation in ecological governance than has been offered by prior unidimensional or purely structural analyses. The specific contributions of this research are threefold. First, a rigorous theoretical integration is achieved by bridging individual-level psychological constructs from TPB with the meso-level social structures emphasized in SNET, thereby addressing a notable gap in the literature on collaborative environmental governance. Second, new mechanistic insight is provided through empirical evidence on the specific pathways, such as the mediating effects of attitude and subjective norm. Finally, enhanced predictive power is expected from the integrated framework, as it combines the explanatory strengths of both theories to deliver a more comprehensive and robust explanation of participation behavior within multi-stakeholder ecological governance contexts. We believe these revisions have effectively addressed all the issues you raised, significantly improving the logical coherence, theoretical rigor, and clarity of the research gap in the literature review. Once again, we are grateful for your constructive comments, which have been instrumental in enhancing the quality of our manuscript. We look forward to your further review. |
| 3. Hypotheses Development and Conceptual Model  Many hypotheses are stated without adequate theoretical justification, and the conceptual model is difficult to interpret. Additionally, constructs such as PO (Perception of Outcome) appear to be used as substitutes for established TPB components, which may lead to conceptual confusion.   Recommendation: Move the hypotheses section directly after the literature review, provide theory-based reasoning for each hypothesis, and revise the conceptual diagram for clarity. If PO is intended to function as a determinant of intention, the authors should explicitly justify this adaptation of TPB using previous studies and clearly explain how PO relates to attitude or intention in this context. | We sincerely appreciate your valuable comments and constructive suggestions on our manuscript, which have significantly helped us improve the rigor and clarity of our research. We have carefully addressed all your concerns regarding the hypotheses development and conceptual model, and the detailed revisions are summarized as follows:  1. As suggested, we have moved the "Hypotheses and Theoretical Model" section immediately after the literature review (renumbered as Section 3.1-3.3 in the revised manuscript). This adjustment ensures that hypotheses are derived from the existing literature and theoretical frameworks, establishing a logical flow from literature synthesis to hypothesis development. Corresponding adjustments to the subsequent sections (e.g., "Methodology" renumbered as Section 4) have been made to maintain the manuscript’s structural consistency.  2. To address the lack of adequate theoretical justification for hypotheses, we have supplemented each hypothesis with systematic reasoning grounded in the Theory of Planned Behavior (TPB) and Social Network Embeddedness Theory (SNET), combined with relevant prior studies and the specific context of ecological co-management at the Lujiangba Coffee Planting Base.  3. To resolve potential conceptual confusion between PO (Perception of Outcome) and established TPB components, we have made explicit clarifications: PO is not a substitute for TPB’s "Behavioral Intention", but an endogenous outcome expectation variable added to adapt to the multi-stakeholder ecological co-management context. It refers to stakeholders’ subjective predictions of ecological, economic, and social outcomes resulting from their participation in ecological co-management, aiming to reflect the impact of behavioral value judgments on participation decisions.  4. To improve the interpretability of the conceptual model (Figure 1 in the revised manuscript), we have revised it with the following adjustments: Exogenous variables (ENW, SNW) are placed on the left, mediating variables (IBA, SN, PBC; TPB core variables) in the middle, and the endogenous variable on the right, clearly distinguishing variable types.  The rewritten third section is as follows. (Pages 9-16; Lines 229-405. Clean version)   1. 3.Hypotheses and theoretical model   3.1 Hypotheses  This section develops hypotheses based on the TPB and SNET, integrating the specific context of ecological co-management at the Lujiangba Coffee Planting Base to systematically deduce the relationships among ENW, SNW, TPB core variables, and Perception of Outcome (PO). Notably, PO is not a substitute for *Behavioral Intention* (a core component of TPB) but an endogenous variable added in this study to adapt to the ecological co-management context. It refers to stakeholders' subjective predictions and perceptions of ecological, economic and social outcomes resulting from their participation in ecological co-management, aiming to accurately reflect the impact of behavioral value judgments on participation decisions. Its association with TPB core variables aligns with the theoretical logic of "Attitude → Outcome Expectation → Behavioral Intention" [34]. This adaptation of TPB is justified by prior studies: scholars have supplemented TPB with outcome perception variables in ecological behavior research to enhance the model's explanatory power for decision-making mechanisms in multi-stakeholder collaboration scenarios [35].  The ENW is conceptualized as the affective bonds and satisfaction derived from participation in ecological co-management [36], whereas the SNW pertains to the informational and advisory influences that shape decision-making behaviors through social interactions [37].  A critical dimension of relational embeddedness, ENW is posited within SNET to strengthen stakeholders’ affective bonds, which in turn may foster an enhanced sense of belonging and responsibility. This strengthened socio-emotional foundation is understood to influence both the attitudes and perceived capabilities of stakeholders engaged in ecological co-management. Simultaneously, SNW, as a principal manifestation of structural embeddedness, operates through the dissemination of information and the reinforcement of social norms, thereby reshaping stakeholders’ cognitive frameworks and behavioral dispositions.  Existing studies have mostly examined the impacts of these two types of networks in isolation, lacking a systematic analysis of their synergistic effects and transmission mechanisms through TPB pathways. Thus, the following hypotheses are proposed:  **H1.** Direct impact of ENW and SNW on TPB core variables  **H1-a:** A statistically significant positive relationship is hypothesized between ENW and the IBA of diverse stakeholders. TPB posits that individual attitudes are driven by both emotional experiences and value judgments [38]. From the SNET perspective, ENW reinforces positive perceptions of behavioral value by establishing stable emotional bonds and improving participation satisfaction [39]. Moreover, satisfied emotional needs connect interactive ties to positive behavioral attitudes, buttressing ENW’s positive effect on IBA [40]. Such cross-domain findings also validate that emotional network bonds strengthen behavioral value perceptions, underpinning the core logic of H1-a.  **H1-b:** ENW is theorized to exert a positive influence on Subjective Norm (SN). Emotional connections amplify the perceived behavioral expectations of reference groups, reinforcing the effect of ENW on subjective norms [41]. Enhanced emotional connectivity within ENW is anticipated to elevate stakeholders' awareness of prevailing behavioral norms among their peers. Particularly in collaboration among coffee farmers, enterprises and the government, emotional trust facilitates the effective transmission of normative information, making stakeholders more sensitive to the behavioral expectations of other actors.  **H1-c:** ENW has a significant positive impact on the PBC of diverse stakeholders. ENW provides emotional support to stakeholders, which can enhance their confidence in their own participation capabilities. Emotional support from social networks significantly improves farmers' perceived control over ecological planting behaviors [42]. The affective reinforcement provided by ENW is expected to bolster individuals' self-efficacy and perceived capacity to engage in ecological governance initiatives.  **H1-d:** SNW is hypothesized to positively affect IBA. As a carrier of information transmission, SNW optimizes stakeholders' cognitive judgments on ecological co-management through the sharing of professional knowledge and governance experience. Given that TPB posits cognitive optimization as a direct antecedent of individual attitudes [13], informational networks can correct biased perceptions of target behaviors, thereby improving individuals' attitudes toward participation [43, 44].  **H1-e:** A significant positive linkage is proposed between the SNW and SN. SNET emphasizes that interactions in structural networks strengthen the binding force of group norms, making individuals more likely to perceive the behavioral expectations of others. Social interactions and information exchange within SNW are posited to amplify individuals' perceptions of normative expectations [44].  **H1-f:** The SNW has a significant positive impact on the PBC of stakeholders. SNW provides abundant information resources and social support, which reduce the costs and risks for stakeholders to participate in ecological co-management, thereby improving their perceived behavioral control [45]. By furnishing informational resources and social support, SNW is theorized to augment stakeholders' confidence in their governance participation capabilities.  **H2:** Direct impact of TPB core variables on behavioral intention  **H2-a:** IBA is hypothesized to exhibit a positive and significant impact on PO. TPB points out that positive individual attitudes drive individuals to form positive predictions of behavioral outcomes [46]. A significant positive correlation exists between positive attitudes toward agricultural ecological behaviors and outcome perceptions [47]. Favorable attitudes toward ecological co-management are anticipated to directly translate into heightened positive perceptions of behavioral outcomes. For example, farmers who recognize ecological co-management are more likely to predict that such behaviors will improve coffee quality and increase income.  **H2-b:** SN has a significant positive impact on PO of stakeholders. Subjective norms reflect individuals' perceptions of others' expectations [48]. When stakeholders perceive that surrounding groups approve of ecological co-management, they tend to predict that the behavior will gain group support and positive outcomes in turn. Stakeholders who perceive strong normative pressure from peers and authorities are more likely to anticipate positive outcomes from participating in ecological co-management.  **H2-c:** PBC is postulated to demonstrate a positive association with PO. Higher PBC means individuals have greater mastery over behaviors, fostering clearer and more positive predictions of behavioral outcomes [49]. For example, farmers with rich planting experience are more likely to predict that ecological planting will achieve an ecological and economic win-win situation.  **H3:** Direct impact of ENW and SNW on behavioral intention  **H3-a:** The ENW directly influences the PO of stakeholders. Trust relationships reduce information asymmetry, increasing stakeholders’ willingness to trust the behavioral outcome experiences shared by others [50]. Affective bonds in ENW facilitate the sharing of authentic experiences of ecological co-management outcomes, enabling stakeholders to form more positive perceptions. For example, trust among coffee farmers in ENW encourages the sharing of real income and ecological benefits from participation, directly shaping others' outcome perceptions.  **H3-b:** The SNW directly influences the PO of stakeholders. Informational exchanges in structural networks provide stakeholders with direct insights into the potential outcomes of ecological co-management, positively shaping their perceptions.  **H4:** Indirect effects of ENW and SNW on behavioral intention.  **H4-a:** ENW is theorized to indirectly shape PO through the mediating roles of IBA, SN, and PBC. ENW first optimizes stakeholders' behavioral attitudes, subjective norm cognition, and perceived behavioral control through emotional bonds, and then further influences their perceptions of behavioral outcomes through the transmission of these three TPB variables.  **H4-b:** SNW is anticipated to exert an indirect influence on PO via the mediation of IBA, SN, and PBC. SNW first reshapes stakeholders' TPB core variables through information transmission and social influence, and then indirectly enhances their positive perceptions of ecological co-management outcomes with the synergistic effect of these variables.  3.2 Theoretical model  Building upon the aforementioned hypotheses, a comprehensive theoretical framework has been constructed to elucidate the underlying mechanisms through which ENW and SNW exert their influence on the participatory behaviors of diverse stakeholders in ecological co-management systems. As demonstrated in Figure 1, the conceptual model integrates multiple dimensions of analysis, thereby offering a systematic representation of the complex interplay among the examined variables.    Fig.1. The conceptual model of this paper.  3.3 Statistical analysis details  3.3.1 Model specification  The Partial Least Squares Structural Equation Modeling (PLS-SEM) employed in this study consists of two sub-models: the measurement model (outer model) and the structural model (inner model). Since all latent variables (IBA, SN, PBC, PO, ENW, SNW) are conceptualized as reflective constructs, the Measurement Model is expressed by the linear Equation (1)  $\text{x}_{\text{j}\text{h}}\text{=}\text{λ}_{\text{j}\text{h}}\text{ξ}_{\text{j}}\text{+}\text{ε}_{\text{j}\text{h}}$ (1)  Where $\text{x}_{\text{j}\text{h}}$ represents the $\text{h}$-th indicator of the $\text{j}$-th latent variable $\text{ξ}_{\text{j}}$, $\text{λ}_{\text{j}\text{h}}$ is the outer loading (correlation between the indicator and the latent variable), and $\text{ε}_{\text{j}\text{h}}$ is the measurement error.  The Structural Model, representing the hypothesized relationships in Figure 2, is defined by the following system of linear Equations (2):  $\text{ξ}_{\text{Endogenous}}\text{=∑}\text{β}_{\text{ji}}\text{ξ}_{\text{i}}\text{+}\text{ζ}_{\text{j}}$ (2)  Specifically, for the core outcome variable (PO), the Equation (3) is:  $\text{PO}\text{=}\text{β}_{\text{1}}\text{IBA}\text{+}\text{β}_{\text{2}}\text{SN}\text{+}\text{β}_{\text{3}}\text{PBC}\text{+}\text{β}_{\text{4}}\text{ENW}\text{+}\text{β}_{\text{5}}\text{SNW}\text{+}\text{ζ}_{\text{PO}}$ (3)  Similarly, the mediating variables are modeled as ( equations (4) -(6)):  $\text{IBA}\text{=}\text{γ}_{\text{1}}\text{ENW}\text{+}\text{γ}_{\text{2}}\text{SNW}\text{+}\text{ζ}_{\text{IBA}}$ (4)  $\text{SN}\text{=}\text{γ}_{\text{3}}\text{ENW}\text{+}\text{γ}_{\text{4}}\text{SNW}\text{+}\text{ζ}_{\text{SN}}$ (5)  $\text{PBC}\text{=}\text{γ}_{\text{5}}\text{ENW}\text{+}\text{γ}_{\text{6}}\text{SNW}\text{+}\text{ζ}_{\text{PBC}}$ (6)  Where $\text{β}$ and $\text{γ}$ represent the path coefficients to be estimated, and $\text{ζ}$ represents the residual variance (structural error).  3.3.2 Estimation and objective function  Unlike covariance-based SEM, PLS-SEM does not optimize a global fit function (such as minimizing the difference between observed and estimated covariance matrices). Instead, it employs an iterative algorithm to maximize the explained variance ($\text{R}^{\text{2}}$) of the endogenous latent variables. The objective function is to minimize the residual variances ($\text{ζ}$) in the structural equations.  3.3.3 Bootstrapping and significance testing  To assess the statistical significance of path coefficients and indirect effects, this study employed the Bias-Corrected and Accelerated (BCa) Bootstrapping method with 5,000 subsamples under a two-tailed testing framework ($\text{α}\text{=0.05}$). The bootstrapping algorithm achieved convergence after three iterations, ensuring the computational stability of the standard error estimates. Specifically, the T-statistics reported in Tables 3 and 4 were calculated as the ratio of the original sample estimate ($\text{O}$) to the standard error ($\text{STDEV}$) derived from the bootstrap distribution ($\text{t}\text{=}\text{O}\text{/}\text{STDEV}$), where $\text{STDEV}$ represents the standard deviation of the 5,000 bootstrap estimates. Furthermore, the significance of mediation path was assessed by calculating the product of coefficients for each bootstrap subsample. The mean of these products served as the point estimate, while their standard deviation across subsamples functioned as the standard error for the T-test.  3.3.4 Calculation of Latent Variable Scores  It is important to clarify that the values of the latent variables used in the structural model were not derived from simple arithmetic means or medians of the observed indicators. Instead, consistent with the PLS-SEM algorithm, latent variable scores were calculated as weighted linear combinations of their respective indicators (Equation (7)):  $\text{ξ}_{\text{j}}\text{=∑}\text{w}_{\text{j}\text{h}}\text{x}_{\text{j}\text{h}}$ (7)  Where $\text{w}_{\text{j}\text{h}}$ represents the outer weights determined by the PLS algorithm to maximize the explained variance, and $\text{x}_{\text{j}\text{h}}$ represents the standardized values of the observed indicators. This method accounts for the varying contribution of each indicator to the construct, offering higher precision than unweighted averaging.  We hope these revisions address your concerns adequately. Thank you again for your rigorous review and invaluable guidance, which have greatly enhanced the quality of our research. |
| 4. Methodology and Sampling  The manuscript does not clearly describe the sampling method, recruitment procedure, or how the participants represent “multiple subjects.” Based on the demographic distribution, the sample appears to consist mostly of residents rather than diverse stakeholder groups. This undermines the claim of multi-subject co-management. Recommendation: Provide explicit details about the sampling strategy and justify how the selected respondents represent the required actors (e.g., farmers, local officials, business owners). Clarify ethical approval and informed consent procedures, which are required for PLOS ONE. If the study targets only a subset of stakeholders, define the study population accordingly. | We sincerely thank the reviewer for this crucial observation. We acknowledge that the previous version lacked explicit details regarding the sampling strategy and the specific breakdown of stakeholder roles, which obscured the "multi-subject" nature of our study. We have made the following major revisions to address these concerns:  1. We have rewritten Section 4.2.2 to explicitly define our stratified purposive sampling strategy. We clarified that the recruitment was structured to ensure representation from four distinct groups: local farmers, enterprise managers, government officials, and community residents. We also detailed the mixed-method recruitment procedure to justify the sampling approach:  4.2.2 Data acquisition and sampling strategy  The formal investigation was conducted from July 2024 to March 2025 using a stratified purposive sampling method to ensure the representation of multiple stakeholders involved in the ecological co-management of the Lujiangba coffee plantation base.  To ensure the diversity of the sample, the target population was divided into four distinct stakeholder groups: (1) Local coffee farmers, who are the direct practitioners of ecological cultivation; (2) Enterprise managers, representing coffee processing factories and estates; (3) Government officials, including staff from the local agricultural bureau and environmental protection station; and (4) Local residents, who are affected by the ecological environment.  To minimize sampling bias, the recruitment procedure employed a mixed-method approach specifically tailored to the characteristics of each stakeholder group. For farmers and residents, face-to-face interviews were conducted in the fields and community centers of Xinzhai and Biton villages, a strategy designed to overcome potential digital barriers. In parallel, the research team visited major coffee estates, such as Xinzhai and Biton, to distribute questionnaires to enterprise managers, while surveys for government officials were administered through local administrative offices. Furthermore, an electronic version of the survey via Wenjuanxing was utilized as an online supplement to effectively reach younger stakeholders and tourists engaged in the coffee culture experience.  A total of 146 questionnaires were distributed. After excluding invalid responses (e.g., incomplete answers or uniform selection), 137 valid questionnaires were obtained, resulting in an effective response rate of 93.8%. (Pages 18-19; Lines 469-489. Clean version)  2. To address the concern that the sample consists "mostly of residents," we have included a detailed breakdown of the respondents' roles based on socioeconomic characteristics in Section 4.2.3:  Crucially, regarding the distribution of stakeholder categories, the sample effectively covers the diverse actors required for the co-management analysis (Figure 2-7). Based on the socioeconomic characteristics of the respondents, the sample comprised 63 local coffee farmers (46.0%), who constitute the core body of ecological practice; 15 enterprise managers (10.9%) from coffee estates and processing factories; 16 government officials (11.7%) involved in local administration and environmental regulation; and 43 local residents and students (31.4%), representing the broader community and educational tourism participants. This balanced distribution validates the study's premise of 'multi-subject' participation and ensures that the perspectives of regulators, producers, and beneficiaries are all adequately represented. (Page 20; Lines 509-518. Clean version)  3. We have added a formal Ethical Statement confirming that the study was conducted in accordance with the Declaration of Helsinki, approved by the Ethics Committee of Baoshan University, and that informed consent was obtained from all participants. |
| 5. Measurement Scales and Variable Operationalization  Some items do not align with validated TPB or SNET measurement scales. A few items appear leading or double-barreled, and the distinction between constructs such as ENW, SNW, and behavioral antecedents is sometimes unclear. Recommendation: Revise the measurement scales to align with validated instruments or justify deviations. Modify vague or leading items. | We appreciate this detailed feedback regarding construct validity and item design. We acknowledge that the phrasing of certain items in the previous version was inconsistent with standard scale development protocols, and the theoretical distinction between network constructs needed further clarification. We have systematically revised the measurement scales and method descriptions to address this:  1. To address the concern regarding "leading" items, we have thoroughly revised Table A3. We converted all interrogative items (e.g., "Do you think...") into standard declarative Likert statements to ensure neutrality.  Example Revision (IBA1): Changed from "Do you believe that effective ecological co-management measures are crucial...?" to "Effective ecological co-management is crucial for protecting the coffee plantation's environment."  Example Revision (PBC1): Changed from "Do you believe you have sufficient capability...?" to "I possess sufficient capability to participate in ecological co-management activities."  2. We identified and simplified items that contained multiple concepts ("double-barreled") to ensure they measure a single construct per item.  Example Revision (PO1): Simplified to focus solely on identity, removing the complex causal link in the original phrasing.  Example Revision (ENW2): Revised to focus exclusively on "emotional connections" rather than mixing interaction and emotion, ensuring a clean measurement of the affective dimension.  3. In Section 4.2.1, we provide a theoretical justification for adapting the standard TPB scales. Given the collaborative nature of the study (co-management), strictly individualistic measures were deemed insufficient. Consequently, we integrated Collective Efficacy into the Perceived Behavioral Control (PBC) scale (Items PBC3, PBC4) and Group Identity/Norms into the Subjective Norms (SN) scale (Items SN4, SN5). These modifications ensure the scales adequately capture the social dynamics inherent in collective action contexts.  All measurement items were assessed using a 5-point Likert scale, where 1 represented 'Strongly Disagree' and 5 represented 'Strongly Agree'. The data were recorded exactly as selected by the respondents without any transformation, truncation, or modification to the original values. regarding data completeness, the final dataset contained no missing values, as incomplete questionnaires were excluded during the data cleaning phase prior to analysis. (Page 18; Lines 464-468. Clean version) |
| 6. Results Interpretation  Although the statistical procedures are appropriate, the interpretation of mediated effects and relationships lacks theoretical depth. The distinction between direct and indirect pathways is described but not meaningfully explained. Recommendation: Expand the explanation of why ENW might influence subjective norms more strongly and why SNW may relate more closely to perceived behavioral control. Connect these interpretations to previous environmental psychology and network studies to strengthen the theoretical contribution. | Thank you for your valuable feedback on our manuscript. We fully acknowledge your comment that the interpretation of mediated effects and the distinction between direct and indirect pathways lacked sufficient theoretical depth, and we appreciate your constructive recommendation to strengthen these aspects by connecting our findings to environmental psychology and social network research. We have thoroughly revised the Results and Discussions section to address these concerns, and the key modifications are summarized below:   1. To clarify why emotional networks (ENW) exert a stronger influence on subjective norms (SN) and why suggestive networks (SNW) are more closely linked to perceived behavioral control (PBC), we integrated foundational theories and empirical literature to elaborate on the underlying mechanisms:   ENW’s stronger impact on SN: Furthermore, the analysis indicates that ENW significantly predicts SN with a stronger path coefficient compared to SNW’s influence on SN. This discrepancy can be theoretically explicated by social identity theory and environmental psychology research: ENW foster in-group cohesion and value alignment through affective bonds (e.g., trust, satisfaction, and shared experiences), which amplify individuals’ sensitivity to normative expectations within the community [55]. In ecological co-management contexts, ENW-derived emotional attachments strengthen stakeholders’ identification with the collective environmental goals, making them more likely to internalize group norms as personal standards [56] For instance, participation in community-led tree-planting events or environmental clean-ups nurtures a sense of “we-ness,” which reinforces the perceived social pressure to engage in pro-ecological behaviors, thus explaining ENW’s stronger impact on SN. (Page 25; Lines 592-602. Clean version)  SNW’s closer association with PBC: The path coefficient from the SNW to PBC is 0.282 (t=3.66, p<0.001), significant at the 0.001 level. This robust association is theoretically grounded in the information-processing perspective of environmental psychology and social network theory: SNW specialize in transmitting practical knowledge, technical guidance and resource-related information that directly enhances stakeholders’ perceived capability to engage in co-management [57]. Unlike ENW, which operates through affective empowerment, SNW reduces perceived behavioral barriers by addressing informational asymmetries and building functional competence [58]. For example, SNW indicators capturing the dissemination of sustainable coffee cultivation techniques or government subsidy policies equip stakeholders with tangible resources and skills, thereby strengthening their confidence in executing ecological management tasks [59]. (Page 26; Lines 628-637. Clean version)   2. We supplemented the manuscript with a dual-process framework to meaningfully explain the difference between direct and indirect effects:   To further elucidate the distinction between direct and indirect pathways, a theoretical framework grounded in dual-process models of decision-making is proposed [61]: This framework delineates two principal mechanisms: (1) Direct effects (ENW→PO and SNW→PO) are characterized by automatic, affect- or information-driven processes that circumvent deliberate cognitive evaluation. In this pathway, pre-existing emotional bonds are leveraged by ENW to elicit immediate pro-intentions, whereas goal-relevant information is utilized by SNW to align intentions without mediation through attitudinal or control-related constructs. (2) Indirect effects (e.g., ENW→IBA→PO, SNW→PBC→PO), in contrast, entail deliberative cognitive processing wherein core constructs of the attitudes, subjective norms and perceived behavioral control, are initially shaped by network influences prior to their impact on behavioral intentions. For instance, indirect influence of ENW on PO is mediated through the fostering of positive attitudes, which reflect a cognitive appraisal of co-management benefits. Similarly, SNW operates indirectly by enhancing perceived behavioral control, defined as a cognitive assessment of one’s capability to perform the behavior [62]. This dual-process distinction is consistent with prior environmental psychology research, which posits that pro-environmental intentions may emerge from both automatic (implicit) and deliberate (explicit) psychological mechanisms [63]. Consequently, the present framework advances a more nuanced understanding of the complementary pathways through which social networks shape participatory behavior in ecological co-management contexts. (Page 30; Lines 719-737. Clean version)   3. We explicitly articulated how these revisions advance existing literature:   The theoretical contributions of this study are threefold. First, a clarification is provided regarding the differential mechanisms through which ENW and SNW shape the constructs of the TPB. Specifically, ENW is found to exert a stronger influence on subjective norms via social identity and affective bonding, whereas SNW is more closely associated with perceived behavioral control through informational support and competence development. This distinction addresses a gap in the extant literature, wherein social networks have frequently been conceptualized as a unidimensional construct. Second, by integrating dual-process models of decision-making with TPB and SNET, a theoretical explanation is advanced for the distinct direct and indirect pathways through which network embeddedness influences participatory intentions. This integrated framework yields a more nuanced understanding of social influence mechanisms within the context of ecological governance. Third, the empirical findings validate the cross-context applicability of social identity theory and self-efficacy theory within agricultural-ecological co-management systems, thereby extending their relevance beyond conventional environmental psychology research domains.  In conclusion, this study contributes to the literature by furnishing empirical evidence on the multidimensional role of social network embeddedness in ecological co-management and by offering a theoretically grounded explanation for the differential effects exerted by emotional versus suggestive networks. The findings carry practical implications for the design of targeted interventions aimed at enhancing stakeholder participation. It is recommended that policymakers and practitioners prioritize the cultivation of emotional bonds to reinforce normative compliance. Concurrently, investments in informational networks, such as training programs and structured policy dissemination, are advised to strengthen stakeholders’ perceived behavioral control. (Pages 32-34; Lines 808-840. Clean version)  We believe these changes significantly enhance the theoretical depth of our results interpretation and strengthen the overall contribution of the study. Thank you again for your insightful comments, which have helped improve the quality and rigor of our work. |
| 7. Discussion, Policy Implications, and Limitations  The discussion mainly repeats the results rather than offering deeper theoretical or practical insights. Policy recommendations are broad and not clearly derived from the findings. Additionally, limitations regarding sample size, generalizability, and measurement issues are not acknowledged. Recommendation: Strengthen the discussion by explicitly linking findings to TPB and SNET, provide evidence-based policy recommendations relevant to ecological co-management in agricultural communities, and include a dedicated limitations section. | Thank you for your insightful feedback on our manuscript. We fully appreciate your comments regarding the need to strengthen the discussion with deeper theoretical insights, refine policy recommendations to be more evidence-based, and include a dedicated limitations section addressing sample size, generalizability, and measurement issues. We have thoroughly revised Section 6 (Conclusions, Policy Implications, and Limitations) to address these concerns, and the key modifications are summarized below:   1. To move beyond mere repetition of results and provide deeper theoretical insights, we have rewritten the Conclusions section to explicitly integrate our findings with the core tenets of the Theory of Planned Behavior (TPB) and Social Network Embeddedness Theory (SNET):   **6. Conclusions and policy recommendations**  6.1 Conclusions  This study advances the integration of the TPB and SNET by empirically elucidating the multidimensional mechanisms through which social networks shape multi-stakeholder ecological co-management intentions within agri-tourism communities. Building upon the TPB’s focus on cognitive antecedents, i.e., attitudes, subjective norms, and perceived behavioral control and SNET’s emphasis on relational embeddedness, it is demonstrated that emotional networks and suggestive networks operate through distinct yet complementary pathways to influence participation intentions. This theoretical extension addresses the historical separation between cognitive and relational perspectives in environmental behavior research.  Specifically, the core proposition of the TPB is validated, confirming that individual behavioral attitudes, subjective norms, and perceived behavioral control serve as key predictors of behavioral intentions, with individual behavioral attitudes emerging as the strongest driver. However, through the integration of SNET, it is further revealed that social networks amplify the explanatory power of the TPB via dual-process mechanisms. First, automatic pathways are identified, wherein emotional networks trigger affective priming and suggestive networks provide cognitive clarity, thereby bypassing deliberate cognitive mediation. Second, deliberative pathways are observed, whereby emotional networks strengthen subjective norms through social identity formation, while suggestive networks enhance perceived behavioral control via informational support.  These findings refine SNET by distinguishing between emotional embeddedness and informational embeddedness. It is shown that emotional networks exert a stronger influence on subjective norms, whereas suggestive networks are more closely linked to perceived behavioral control, a nuanced distinction that extends the understanding of how different network functions shape the constructs of the TPB.  From a practical perspective, the results highlight that intrinsic motivation and self-efficacy outweigh normative pressure in driving ecological co-management intentions within agricultural settings. This underscores the need to prioritize attitude change and capability building alongside interventions based on social norms. By bridging the TPB and SNET, a unifying framework is provided for explaining multi-stakeholder environmental behavior, demonstrating that social networks do not merely supplement cognitive factors but actively reshape them through affective and informational mechanisms. This study investigated the behavioral driving mechanisms of multi-stakeholder ecological co-management in a China’s coffee-producing area by integrating the TPB and SNET. The results demonstrate that both ENW and SNW significantly enhance stakeholders’ participation intentions through direct and indirect pathways. Specifically, higher levels of ENW are associated with stronger IBA, SN, and PBC by fostering emotional bonds and collective identity. Meanwhile, SNW is positively linked to IBA, SN, and PBC, potentially through pathways of information dissemination. It is worth noting that, descriptively, both IBA and PBC exhibited larger path coefficients regarding participation intentions compared to SN, highlighting the importance of intrinsic motivation and self-efficacy in ecological co-management.  This study advances the TPB by incorporating SNET, revealing how social networks shape behavioral intentions beyond traditional cognitive factors. It bridges a critical gap in multi-stakeholder governance research by systematically analyzing relational embeddedness rather than focusing solely on structural network properties. (Pages 32-33; Lines 785-830. Clean version)   2. We have revised the policy recommendations to be directly derived from our empirical findings, focusing on actionable interventions tailored to agricultural communities’ ecological co-management needs:   6.2 Policy recommendations  Guided by empirical findings on the differential mechanisms of emotional networks and suggestive networks, along with their targeted effects on the constructs of the Theory of Planned Behavior, evidence-based and context-specific policy recommendations are proposed. These recommendations are tailored to the ecological co-management needs of agricultural communities, moving beyond broad governance frameworks toward actionable interventions aligned with both theoretical and statistical insights.  To strengthen normative internalization and emotional commitment, the reinforcement of emotional networks is recommended. Given the stronger influence of emotional networks on subjective norms through social identity formation, policy interventions should prioritize the building of affective bonds to enhance normative compliance. Community-based emotional bonding programs can be designed, featuring regular ecology-themed collective activities, such as joint forest restoration and communal composting projects, which integrate environmental stewardship with social interaction. Additionally, affective recognition systems, such as village-level ecological stewardship champions programs, can be implemented to publicly honor active participants through ceremonies or local media. Shared narrative building through local storytelling campaigns, including oral histories of environmental conservation and community-led documentaries, is also encouraged to cultivate emotional commitment to communal ecological goals.  To enhance individual behavioral attitudes and perceived behavioral control, the optimization of suggestive networks is advised. Leveraging the robust association of suggestive networks with perceived behavioral control and its significant effects on individual behavioral attitudes, policies should focus on informational support and capacity building. Multi-channel, practical information dissemination can be established through an ecological co-management information hub that integrates digital platforms and traditional channels to deliver targeted content. This includes data on local ecological benefits to reshape attitudes toward co-management, as well as step-by-step guides on sustainable cultivation techniques and subsidy application processes to address informational asymmetries. Furthermore, opinion leader engagement can be promoted by training local coffee farmers, enterprise managers, and village leaders as ecological ambassadors to disseminate information and model pro-environmental behavior. Practical skill-building workshops, offering hands-on training on ecological management tailored to agricultural stakeholders’ needs, are also recommended to directly enhance perceived behavioral control.  Finally, the establishment of a network-centered co-management mechanism is proposed to integrate the effects of emotional and suggestive networks into governance structures. Stakeholder network mapping should be conducted to systematically identify existing emotional and suggestive networks, key actors, and areas where networks are weakest. Participatory decision-making forums can be created to hold regular, inclusive meetings that combine social interaction with information exchange, ensuring that both emotional bonds and practical knowledge inform policy design. Moreover, cross-sectoral network coordination should be mandated to align community activities focused on emotional networks with capacity-building efforts focused on suggestive networks, thereby creating a synergistic system that addresses both affective and cognitive barriers to participation. (Pages 33-35; Lines 854-896. Clean version)   3. We have expanded the Limitations section to systematically acknowledge issues related to sample size, generalizability, and measurement, as well as other methodological considerations:   6.3 Limitations  Several limitations of this study should be acknowledged. First, the generalizability of the findings may be constrained by the relatively small sample drawn from a single coffee-producing region, which limits the applicability of the results to agricultural communities with distinct socioeconomic or geographical characteristics. Second, the measurement of emotional and suggestive networks relies on self-reported data, which could be influenced by social desirability bias, and the cross-sectional design precludes the establishment of causal relationships between network mechanisms and behavioral intentions. Third, while relational embeddedness is examined, structural properties of the networks are not accounted for, nor are potential moderating effects of individual differences across stakeholder types. Addressing these limitations in future research would strengthen the theoretical and practical contributions of this line of inquiry. (Page 35-36; Lines 897-908. Clean version)  We believe these changes significantly enhance the theoretical depth of the discussion, strengthen the practical relevance of policy recommendations, and provide a transparent assessment of the study’s limitations. Thank you again for your constructive feedback, which has greatly improved the rigor and impact of our work. |
| 8. Abstract  The abstract is too general and does not clearly communicate the context, theoretical approach, methodology, or major findings.   Recommendation: Improve the abstract by briefly stating (a) the ecological problem in the region, (b) the theoretical model (TPB + SNET) and its rationale, (c) sample size and analysis method, and (d) the key direct/indirect effects. A clear statement of contribution should be added to the final sentence. | Thank you for your valuable feedback regarding the abstract. We have revised it to clearly address all the points you raised. Below is the revised abstract, structured to incorporate your recommendations:  **Abstract**  The coffee-growing areas in the Gaoligong Mountains of China face ecological challenges including soil erosion and water pollution from traditional processing methods. To analyze the drivers of stakeholder participation in addressing these issues, this study integrates the Theory of Planned Behavior (TPB) and Social Network Embeddedness Theory (SNET), which together explain how individual cognitions and social structures shape cooperative behavior. Data from a stratified survey of 137 stakeholders were analyzed using PLS-SEM. The results demonstrate that (1) Both emotional networks (ENW) and suggestive networks (SNW) have significant direct effects on participation intention. (2) Indirectly, ENW enhances intention by strengthening individual behavioral attitude, subjective norm, and perceived behavioral control through emotional bonds and identity. (3) SNW improves intention primarily by boosting perceived behavioral control through information dissemination. And (4) the core TPB constructs (attitude, norm, control) are confirmed as key mediators. This paper contributes a validated integrated framework that elucidates the social-psychological pathways for fostering effective ecological co-management.  The revised version now includes:  (a) A clear statement of the regional ecological problem in the opening sentence.  (b) Explanation of the TPB + SNET theoretical model and its rationale.  (c) Specification of the sample size and analytical method (structural equation modeling).  (d) Summary of key direct/indirect effects of emotional and suggestive networks.  (e) A final sentence stating the study’s contribution to theory and practice.  We believe these changes provide a clearer, more structured abstract that aligns with your suggestions. Thank you again for your constructive comments. |
| 9. Language and Presentation   The manuscript requires substantial editing for grammar, clarity, and readability. Several sentences are unclear or repetitive. Figures and demographic tables should be simplified or moved to an appendix.  Recommendation: A thorough English language revision is recommended before resubmission. | We sincerely appreciate your valuable comments on the language and presentation of our manuscript. We fully agree with your observation that the manuscript requires substantial editing to enhance grammar, clarity, readability, and to address unclear or repetitive sentences, as well as to optimize figures and demographic tables. We have carefully followed your recommendations and conducted a comprehensive revision of the entire manuscript’s language. The key modifications are detailed as follows:  First, regarding the enhancement of academic rigor, we have refined the terminology throughout the manuscript to ensure consistency and precision in line with the conventions of our research field. We have replaced informal expressions with more academic wording, particularly in the literature review, theoretical framework, and results discussion sections, to strengthen the manuscript’s scholarly nature.  Second, we have significantly improved the logical coherence between sentences and paragraphs. We have added appropriate transitional words and phrases (e.g., "specifically," "furthermore," "consequently") to clarify the causal, sequential, and associative relationships between ideas. Additionally, we have restructured disjointed sentences and removed repetitive content to ensure a smooth and logical flow of arguments.  Third, we have thoroughly checked and corrected tense inconsistencies across the manuscript. We have standardized the use of tenses in accordance with academic writing norms: the simple present tense is used for theoretical statements, established facts, and the description of the conceptual model; the simple past tense is used for the presentation of research methods, data collection, and analysis processes; and the present perfect tense is used to indicate completed revisions and their impacts on the manuscript.  Furthermore, we have simplified the figures (including the conceptual model and demographic charts) by removing redundant elements and optimizing visual presentation to improve clarity.  We have conducted a thorough English language revision of the entire manuscript, including grammar, syntax, and phrasing, to meet the standards of academic publication. We believe these revisions have significantly improved the overall quality, clarity, and readability of the manuscript.  Thank you again for your insightful comments. |
|  | Dear Reviewer,  We would like to thank you again for the time you spent reading our manuscript and providing the helpful comments above. As you can see, we have make every effort to address them. We hope that you will be satisfied with our responses to these comments.  Thank you for your work. |

| **Response to Reviewer 2** | | |
| --- | --- | --- |
| **Reviewer 2’s comments** | | **Authors’ response** |
| The review is uploaded as an attachment, for improved legibility. In summary: - statistical significance analysis does not account for multiple hypothesis testing and associated alpha inflation (I am hesitant about Question 2 above, but this is not a fatal flaw);  - some conclusions or statements could be reformulated to avoid implying definitive empirical inference about causality, or to clearly separate empirical results from theoretical interpretation or discussion, or to avoid statements about comparisons that were not validated statistically;  - additional technical details are necessary to allow better understanding and reproduction of the results.  All these issues can be corrected and I do not see other obstacles to publication; the study is conducted thoroughly and well-written up. | | Dear Reviewer,  Thank you very much for the time you spent carefully reading and reviewing our paper.  We would like to express our sincere gratitude to you for the positive evaluation of our study's thoroughness and writing quality. Your constructive feedback regarding statistical rigor, causal inference, and technical transparency has been invaluable in strengthening the scientific integrity of our work. We have carefully addressed each of your concerns by implementing more conservative statistical tests, refining our theoretical claims, and enhancing the methodological documentation to ensure our study meets the high standards for publication in PLOS ONE.  To address the concern regarding "alpha inflation" from our 17 hypothesis tests, we have added a sensitivity analysis in Section 5.5. We applied the Bonferroni correction to set a more stringent significance threshold (p<0.0029). While our core structural paths remain robust under this conservative standard, we have transparently updated the manuscript to categorize results within the 0.003 to 0.05 range as "suggestive evidence." This adjustment ensures that our findings are interpreted with appropriate statistical caution.  We fully concur with the your point that SEM results represent statistical associations rather than definitive causality. Accordingly, we have conducted a comprehensive linguistic revision throughout the paper. Terms implying direct causation, such as "influence" or "predict," have been replaced with relational descriptors like "is associated with" or "consistent with theoretical propositions." Furthermore, we have explicitly addressed the inherent limitations of cross-sectional data in establishing causality in the revised Section 6.3.  In line with the requirements for reproducibility, we have added Section 3.3 to provide the explicit mathematical formulas for our measurement and structural models, along with specific PLS-SEM algorithm settings and bootstrapping parameters. Additionally, we have provided the original Chinese questionnaire and the complete SmartPLS iteration report in the Supporting Information files. These additions provide the necessary technical depth for peer researchers to verify and build upon our results.  In summary, we believe these systematic revisions have significantly enhanced the precision and transparency of our research. The inclusion of more rigorous statistical thresholds and a more nuanced interpretation of causal relationships ensures that our conclusions are firmly grounded in the empirical evidence. We once again thank the reviewer for your meticulous guidance, which has played a crucial role in improving the quality of this manuscript. We hope these changes meet your expectations. |
| The study aims to provide insight into the behavioral mechanisms explaining why people engage in ecological co-management (as stakeholders), with the hope of shaping policy to encourage such desirable behavior. Specifically, the intention to participate in such activity (PO), treated as a latent variable, is hypothesized to be directly influenced by 5 other latent variables (ENW, SNW, IBA, SN, PBC), conceptually rooted in the theories of planned behavior (TPB) and of social network embeddedness (SNET). Two of these variables (ENW, SNW) are also hypothesized to (positively) influence the other 3 (IBA, SN, PBC), thus exerting also indirect influence on the latent variable of interest (PO). The Authors proceed to test their hypotheses by means of Structural Equation Modelling (SEM), where observed variables, related to the latent variables, are answers to questions disseminated among stakeholders (residents, managers, government officials, NGOs) in Lujiangba coffee plantation base in Yunnan, China.  The dataset consists of 137 (93.8%) participants' valid responses to a questionnaire consisting of 23 questions (incl. 3 questions representing PO), with responses on a Likert scale from 1 to 5 (question IBA2 seems to be an open question, but in the dataset it appears to follow Likert scale). Each question and answer is assigned to exactly one latent variable.  Answers to questions corresponding to the same latent variable show sufficiently high correlation, and to different latent variables – appropriately low correlation. Answers associated with the variable of primary interest, PO, exhibit elevated correlations with answers corresponding to the other latent variables. A participant’s answers to questions related to the same latent variable are presumably averaged to obtain estimate of the value of that latent variable for that participant.  The authors then iteratively estimate the hypothesized model parameters – path coefficients (only those presumed a priori to differ from zero), which determine relationships between the investigated variables – of their (presumably linear) model. With Structural Equation Modelling (SEM), parameters are typically optimized with respect to covariances between all the latent variables, rather than with respect to prediction of a selected target (response, independent) variable from other available (explanatory) variables. The hypothesized model is fitted to all available data at once and is not validated on an out-of-sample test set, as would be more typical of a primarily predictive regression model. Goodness-of-fit of the whole model is not evaluated, significance is only assessed for its individual coefficients, using BiasCorrected and Accelerated (BCa) bootstrapping to obtain a T statistic for every coefficient. | | We would like to express our heartfelt gratitude to you for the extremely professional and thorough assessment of our manuscript. We are honored by your recognition that the study is well-conducted and that the issues identified are correctable obstacles to publication. Your insightful feedback on our research design and statistical rigor has provided us with a clear roadmap for improving the scientific integrity of this work, and we have implemented comprehensive revisions to address each of your concerns.  We sincerely appreciate the your accurate and comprehensive summary of our study’s objectives and methodological framework. We confirm that your description of the data collection process in the Lujiangba coffee plantation and the application of Structural Equation Modeling (SEM) to test the integration of TPB and SNET is entirely correct. Regarding the measurement details, we confirm that all 23 items were indeed measured on a 5-point Likert scale. While the reviewer noted the use of averages to estimate latent variable values, we would like to clarify that we employed the PLS-SEM algorithm to calculate latent variable scores as weighted linear combinations of their respective indicators. This method accounts for the varying contribution of each indicator, offering higher precision than unweighted averaging.  In response to your concerns regarding the risk of alpha inflation due to multiple hypothesis testing, we have introduced a dedicated Section 5.5 in the revised manuscript. We conducted a post-hoc sensitivity analysis using the Bonferroni correction, setting an adjusted significance threshold of approximately 0.0029. Under this stringent criterion, seven core direct pathways—including the relationship between attitude and outcome perception—remain statistically robust. While other paths yielded p-values within the standard 0.05 range, we now transparently categorize them as suggestive evidence to ensure a cautious interpretation of the data.  Furthermore, we have systematically refined the language throughout the manuscript to address the concerns regarding causal inference. We have replaced deterministic terms such as "influence" or "predict" with relational descriptors like "associated with" or "consistent with theoretical propositions". We have also introduced a new Section 6.3 to explicitly state that the cross-sectional nature of our data precludes the determination of definitive causality, ensuring that our empirical results are clearly separated from theoretical interpretations.  To satisfy the requirements for reproducibility, we have added Section 3.3 to document the explicit mathematical formulas and estimation settings used in our analysis. This includes the objective function for maximizing explained variance and the detailed T-statistic calculations derived from the bootstrap distribution. We have also updated our data repository to include the original Chinese questions and the complete SmartPLS iteration report. We believe these systematic revisions have significantly enhanced the precision and transparency of our research, and we once again thank you for your meticulous guidance. |
| GENERAL COMMENTS | |  |
| Overall the manuscript is well-written, with good structure. It is clear that the Authors have dedicated appropriate effort to conduct and present a thorough analysis. Most if not all statistical procedures expected with Structural Equation Modelling have been conducted, though some additional clarification or revision would be desirable.  Considering the topic and domain of application of the study, it is particularly important to keep in mind the wide, interdisciplinary audience of PLOS ONE, potentially unfamiliar with the presented methods, as well as that Structural Equation Modelling (SEM) is not free from controversy, or at least misunderstandings. It involves a degree of arbitrariness in model construction, and combined with idiosyncrasies of questionnaire design, even if well-executed and providing novel insights, it is still advisable to remain conservative with respect to accepting it as conclusive evidence of general principles, especially in context of causality. Deciphering human behavior is an inherently difficult and elusive task, so a (greater) degree of reservedness when drawing conclusions is to be expected, and perfectly acceptable.  The remainder of this review is split in three parts:   A. Main concerns (required revisions) B. Request for additional details C. Optional suggestions | | We sincerely appreciate your positive evaluation of our manuscript's structure and the thoroughness of our analysis. We are particularly encouraged by your recognition of the effort dedicated to conducting the statistical procedures expected in Structural Equation Modeling (SEM).  We fully concur with your insightful observations regarding the interdisciplinary nature of PLOS ONE and the inherent complexities of using SEM to decipher human behavior. We acknowledge that model construction and questionnaire design involve nuanced decisions that require a conservative approach when drawing broader conclusions, particularly concerning causality. In response to your guidance, we have implemented several revisions to adopt a more reserved and scientifically rigorous tone throughout the manuscript.  Specifically, we have systematically refined our language to avoid implying definitive empirical inference about causality, moving toward a more relational and theoretical interpretation of the statistical associations found. We have also introduced a dedicated limitations section to explicitly discuss the boundaries of our research design and the potential for alpha inflation in multiple hypothesis testing.  Furthermore, to ensure our methodology is accessible and reproducible for the diverse audience of PLOS ONE, we have provided additional mathematical formulas and technical details regarding our estimation processes and bootstrapping procedures. We believe these adjustments address your concerns regarding the elusiveness of behavioral research while maintaining the novelty of our insights.  Thank you once again for your meticulous review and for providing a clear roadmap through parts A, B, and C to elevate the quality of our work. We hope the subsequent detailed responses and manuscript revisions meet your expectations. |
| A. MAIN CONCERNS  The main concerns in this review pertain to two aspects: assessment of statistical significance in the presence of multiple-hypotheses testing, and formulation of conclusions drawn from the data. | |  |
| **A1. Potential overestimation of statistical significance**  The Authors tested 17 (sub)hypotheses, each with a separate Student’s T-test, each at the 5% significance level, to assess whether each of the 11 estimated model parameters (characterizing the hypothesized direct relationships between latent variables) differ significantly from zero, and whether the 6 additional indirect paths are also significant (it is not entirely clear what specific random variable is being tested in the latter 6 tests; presumably the product of coefficients comprising the tested path). The Authors clearly indicate the p-value and T-statistic for each individual test separately, which allows the Readers to make their own calculations and independent inference at any level of significance, should they be inclined to do so. Nevertheless, when discussing stated conclusions, it is important to consider the level of significance also in context of the whole experiment; especially considering that at least some, if not all, of the hypotheses are related to each other. The experiment-wise Type-I error rate (statistical significance level for the whole model), that is, the probability that at least one of the significant test results is incorrect (at least one of the coefficients confirmed to be significant is actually not) is considerably higher than 5%.  To illustrate the point, let us assume for simplicity, just for a moment, that all 17 tests give a significant result, each with 5% probability that its result is incorrect. Then (assuming the tests are independent; which they may not be, here, but let us set that aside for now) the probability of “an undesirable outcome”, undermining the model in any way, i.e. of at least one test giving an incorrectly significant result (indicating a relationship between variables exists and is significant, when in reality it is not), equals 100% – probability of “the desirable outcome” that all tests give a correctly significant result, which is 100% – 95% ^ 17 = 58%. This 58% is the significance level for the whole experiment (this marked increase from 5% is often referred to as ‘alpha inflation’). It does not matter how many tests actually come out significant, because the significance threshold may be determined prior to the experiment.  When discussing levels of significance or confidence, many Readers may expect that it is the overall, experiment-wise error rate that is controlled at the level stated in the paper. Again, to their credit, the Authors have not made such misleading claims and have in fact clearly and unambiguously stated all statistics. But with respect to the conclusions drawn from these statistics, to have 95% confidence in the formulation of the model as a whole, with all its coefficients, requires application of a more conservative threshold than 5% in all the individual tests.  Probably the simplest way to address the issue is to apply the Bonferroni correction to the significance level of every individual test (that is, divide the significance level by the number of conducted tests), and re-evaluate the significance of each hypothesis. Some would still remain significant, but many would not. The Bonferroni correction may be too conservative in this context, so if the Authors wish, they could explore other alternatives (for example, Holm's step-down procedure). But I would be uncomfortable leaving it as it is, without at least explicitly discussing the issue, for the benefit of less statistically-inclined Readers. Especially considering the rather strong statements with respect to drawn conclusions. | | We extend our sincere gratitude for this statistically rigorous observation. We fully acknowledge that testing multiple hypotheses (17 paths) inherently increases the experiment-wise error rate (alpha inflation), and that relying solely on a conventional $\text{p}\text{<0.05}$ threshold may inadvertently overstate the model's certainty. In accordance with your recommendation, we have introduced a dedicated section, Section 5.5 Sensitivity analysis of statistical significance, to explicitly discuss this issue and ensure full transparency for the readers.  Specifically, our revision incorporates the following three-step approach:  Firstly, we established a more conservative significance benchmark using the Bonferroni correction. Given the 17 paths tested under an overall significance level of 0.05, the adjusted threshold for individual path significance was calculated to be approximately 0.0029 ($\text{0.05/17}$).  Secondly, a re-evaluation of our structural model against this stringent criterion confirms the high robustness of our core findings. Seven out of the eleven direct structural paths—including critical relationships such as $\text{IBA}\text{→}\text{PO}$, $\text{SNW}\text{→}\text{PBC}$, and $\text{ENW}\text{→}\text{SN}$—yielded p-values below 0.0029. We have emphasized in Section 5.5 that these fundamental links remain statistically significant even under the most rigorous error control.  Thirdly, we have refined the interpretation of the remaining paths (including indirect mediation effects) that fall within the $\text{0.003<}\text{p}\text{<0.05}$ range. In Section 5.5, we transparently categorize these findings as "suggestive evidence" within the standard 95% confidence interval. This nuanced approach allows us to acknowledge the potential for alpha inflation while retaining theoretically valuable insights that are essential for an exploratory study of this nature.  We believe these additions strengthen the statistical integrity of the manuscript. Thank you again for your constructive guidance, which has greatly improved the rigor of our work.  The revised Section 5.5 is as follows:  5.5 Sensitivity analysis of statistical significance  To address the potential issue of alpha inflation arising from multiple hypothesis testing (comprising 11 direct and 6 indirect paths), a post-hoc sensitivity analysis was conducted using the Bonferroni correction. With an overall significance level set at 0.05, the adjusted threshold for individual path significance was established at approximately 0.0029 (0.05/17). The results, as summarized in Table 3, indicate that seven core direct pathways demonstrate high robustness under this conservative criterion: $\text{IBA}\text{→}\text{PO}$ ($\text{p}\text{<0.001}$), $\text{SNW}\text{→}\text{PBC}$ ($\text{p}\text{<0.001}$), $\text{ENW}\text{→}\text{SN}$ ($\text{p}\text{=0.001}$), $\text{ENW}\text{→}\text{IBA}$ ($\text{p}\text{=0.001}$), $\text{SNW}\text{→}\text{PO}$ ($\text{p}\text{=0.001}$), $\text{SNW}\text{→}\text{IBA}$ ($\text{p}\text{=0.002}$), and $\text{PBC}\text{→}\text{PO}$ ($\text{p}\text{=0.002}$). These relationships represent the fundamental structure of our theoretical model and remain statistically significant even under the most stringent error control.  Although four additional direct paths ($\text{ENW}\text{→}\text{PO}$, $\text{ENW}\text{→}\text{PBC}$, $\text{SN}\text{→}\text{PO}$, $\text{SNW}\text{→}\text{SN}$) and five indirect mediation paths (see Table 4) yielded p-values within the range of 0.003 to 0.05, they are retained as meaningful findings in this study. This decision is supported by the fact that the Bonferroni correction is often considered overly conservative for structural equation modeling (SEM), where paths are theoretically interdependent rather than isolated. Furthermore, given the exploratory nature of applying SNET to ecological co-management, prioritizing the avoidance of Type II errors is essential to capture emerging social mechanisms. Consequently, we interpret these results with transparency: while the core structural links are exceptionally robust, the secondary effects and specific mediation mechanisms should be viewed as suggestive evidence within the standard 95% confidence interval, interpreted within the broader context of experiment-wise error rates. (Pages 30-31; Lines 739-760. Clean version) |
| **A2. Potential overstatement of conclusions (esp. w.r.t. causality)**  Causality is notoriously difficult to establish, and in Structural Equation Modelling, it tends to be assumed from theory. Here, for example, it is assumed that SNW and ENW influence IBA, SN and PBC rather than vice versa. The empirical results alone describe only the strength of the association between these variables, but not its direction. Ignoring the theory behind the experiment, one could construct a similar but different model, in which it is the IBA, SN and PBC that influence PO directly or indirectly through SNW and ENW. Such a model would receive exactly the same empirical support with the methods presented in this paper (if the Authors disagree, please elaborate and expand on the methodology; cf. also section B of this review). Therefore, any conclusions about cause and effect in this study are based on theoretical assumptions rather than empirical evidence.    As an example, let us consider the statement from line 381:  *"The structural equation modeling results reveal that a statistically significant positive relationship exists between ENW and IBA (t=3.27, p=0.001), demonstrating that emotional networks exert a substantial influence on the formation of positive attitudes toward ecological co-management. "*  Only the first part of the sentence reflects the empirical results, while the later part (from “demonstrating that”) reflects the theoretical assumption. The latter part of the statement is not proven or implied by the former part.  Likewise, on line 506:  *“The analysis has demonstrated that both types of social networks exert significant positive effects on the key TPB constructs.”*  "the affective components captured by the ENW2 measurement item have been demonstrated to 455 facilitate the formation of favorable cognitive evaluations regarding co-management behaviors."    On line 533, in turn, there is a **comparison**:  “*both IBA and PBC exhibit stronger effects on stakeholders’ participation intentions than SN*"  The strength of the relationship between SN and PO was not compared, statistically, to the strength of the relationship between PBC and PO, or IBA and PO, so this statement should be framed as exploratory analysis or observation to be further investigated in the future, rather than a definitive conclusion. Alternatively, an additional, explicit statistical test, or rather series of tests (Tukey’s procedure could perhaps be applicable), may be conducted to establish whether the differences between coefficients are significant – but please bear in mind the earlier remarks about alpha inflation with multiple testing.    As mentioned at the beginning, the nature of the experiment does not, strictly speaking, involve **prediction**, or in particular, evaluation of predictive performance. Therefore the following statements are somewhat controversial at best (esp. the third one, as it implies statistical comparison of predictive power):   - "" Furthermore, the analysis indicates that ENW significantly predicts SN ( t=3.196, p=0.001)." (line 388) - The analytical outcomes reveal that IBA serves as the most potent predictor of PO (t=3.654, p<0.001), a finding that strongly corroborates the central tenet of TPB regarding attitude-behavior consistency. The measurement robustness of the IBA construct (AVE=0.701) further reinforces the validity of Hypothesis H2-a.   Furthermore, consistent with the foundational principles of TPB, all three mediating variables were found to be significant predictors of participation intentions.  On line 528, we read:  *"The results demonstrate that (…)* *ENW strengthens IBA, SN, and PBC by fostering emotional bonds and collective identity. Meanwhile, SNW improves IBA, SN, and PBC through information dissemination and social influence."*  Firstly, again, the results do not demonstrate whether SNW improves IBA, SN and PBC, or whether IBA, SN and PBC improve SNW. Secondly, the experiment investigates whether there is any relationship between SNW and IBA, SN and PBC, it does not determine the **mechanism** of that relationship. It examines "whether", not "how", SNW (or ENW) – according to theory – may influence the other three variables. Arguably, this is the section in which general conclusions are presented, with a degree of flexibility with respect to implications, discussion, etc., but still, it would be desirable to draw a clearer distinction between what directly follows from the empirical analysis, and what constitutes a wider, general interpretation and discussion of potential (not conclusively determined) mechanisms.  A similar example is found on line 402:  "*The analytical results further demonstrate that SNW exerts a significant positive effect on IBA (t=3.132, p=0.002). This relationship can be attributed to the dual mechanisms of information dissemination and social influence operating within suggestive networks, where inputs from community opinion leaders substantially shape participants' cognitive frameworks."*  In this case (aside from implied causality), arguably, the phrasing "can be attributed to" seems to indicate an interpretation, but a more explicit distinction would be preferable.    In general, it is good that the authors discuss possible mechanisms. However, it is also desirable to **separate empirical findings from their interpretation and discussion** more clearly, so that even Readers who do not read the paper in full still have appropriate understanding of what constitutes the empirical findings, and what constitutes their theoretical context. This doesn’t have to be a spatial separation, just slightly different wording.    On another note – a challenge with drawing conclusions in this and similar studies lies also with the definition (in particular, scope) of latent variables, and how much of their interpretation is actually captured by the observed variables. Latent variables are abstract concepts, which are only imperfectly expressed through the observed variables, and in the interpretation of results, one must bear that reservation in mind. The interpretation of results cannot rely on a wider understanding of the latent concept that the specific observed variables allow. | | First and foremost, we would like to express our sincere gratitude for your insightful and meticulous critique regarding the overstatement of conclusions and the assumptions of causality in our study. Your observation that Structural Equation Modeling (SEM) relies on theoretical directionality rather than empirical proof of causality is well-taken.  We have carefully revised the manuscript to ensure a clear distinction between empirical statistical associations and theoretical interpretations. Specifically, we have replaced causal language (e.g., "exert effect," "influence," "predict") with relational language (e.g., "associated with," "consistent with") and we have added a robust limitations section to address the boundaries of our latent variables and research design.  Below is the point-by-point detail of the specific text revisions:  1. To address the concern that our results describe associations rather than proven directional causes, we have adjusted the wording throughout the manuscript to reflect this distinction.  Regarding Pages 25; Lines 739-760. Clean version  Original: "The structural equation modeling results reveal that a statistically significant positive relationship exists between ENW and IBA (t=3.27, p=0.001), demonstrating that emotional networks exert a substantial influence on the formation of positive attitudes toward ecological co-management."  Revision: We removed the definitive phrase "demonstrating that" and we reframed the finding as being consistent with theoretical propositions.  Revised Text: "The structural equation modeling results reveal a statistically significant positive relationship between ENW and IBA ($t=3.27,p=0.001$). This finding is consistent with the theoretical proposition that emotional networks facilitate the formation of positive attitudes toward ecological co-management."  Regarding Pages 31; Lines 768-769. Clean version  Original: "The analysis has demonstrated that both types of social networks exert significant positive effects on the key TPB constructs."  Revision: We changed "demonstrated... exert effects" to the more statistically accurate "indicates... associations."  Revised Text: "The analysis indicates that both types of social networks have significant positive associations with the key TPB constructs."  Regarding Pages 28; Lines 680-682. Clean version  Original: "the affective components captured by the ENW2 measurement item have been demonstrated to facilitate the formation of favorable cognitive evaluations regarding co-management behaviors."  Revision: We replaced the definitive "demonstrated to facilitate" with a more cautious "appear to coincide with."  Revised Text: "Specifically, the affective components captured by the ENW2 measurement item appear to coincide with the formation of favorable cognitive evaluations regarding co-management behaviors."  Regarding Pages 25; Lines 593-594. Clean version  Original: "Furthermore, the analysis indicates that ENW significantly predicts SN (t=3.196, p=0.001)."  Revision: We replaced the predictive term "predicts" with "positively associated with."  Revised Text: "The analysis indicates that ENW is significantly and positively associated with SN ($t=3.196,p=0.001$)."  2. To address the critique regarding the lack of explicit statistical tests for comparing coefficients, we have reframed these statements as descriptive observations or exploratory findings.  Regarding Pages 34; Lines 847-849. Clean version:  Original: "both IBA and PBC exhibit stronger effects on stakeholders' participation intentions than SN"  Revision: We now clarify that these are descriptive comparisons of path coefficients rather than statistically tested differences in strength.  Revised Text: "It is worth noting that, descriptively, both IBA and PBC exhibited larger path coefficients regarding participation intentions compared to SN, highlighting the importance of intrinsic motivation and self-efficacy in ecological co-management."  Regarding Pages 27; Lines 646-647. Clean version:  Original: "The analytical outcomes reveal that IBA serves as the most potent predictor of PO (t=3.654, p<0.001), a finding that strongly corroborates the central tenet of TPB regarding attitude-behavior consistency."  Revision: We replaced "most potent predictor" with "strongest correlate" and we specified it is within the conceptual framework to avoid implying absolute predictive power.  Revised Text: "The analytical outcomes reveal that IBA serves as the strongest correlate of PO ($t=3.654,p<0.001$) within the conceptual framework, a finding that strongly corroborates the central tenet of TPB regarding attitude-behavior consistency."  Regarding Pages 31; Lines 776-778. Clean version:  Original: "Furthermore, consistent with the foundational principles of TPB, all three mediating variables were found to be significant predictors of participation intentions"  Revision: We replaced "predictors" with "significantly associated with" to align with the relational nature of the data.  Revised Text: "Furthermore, consistent with the foundational principles of TPB, all three mediating variables were found to be significantly associated with participation intentions."  3. To address the concern about "how" vs. "whether" a relationship exists, we have clarified that proposed mechanisms (like information dissemination) are theoretical interpretations of the statistical links rather than direct empirical proofs of the process.  Regarding Pages 34; Lines 844-847. Clean version:  Original: "Specifically, ENW strengthens IBA, SN, and PBC by fostering emotional bonds and collective identity. Meanwhile, SNW improves IBA, SN, and PBC through information dissemination and social influence."  Revision: We changed causal verbs ("strengthens/improves") to relational ones and we framed the mechanism as a potential pathway.  Revised Text: "Specifically, higher levels of ENW are associated with stronger IBA, SN, and PBC by fostering emotional bonds and collective identity. Meanwhile, SNW is positively linked to IBA, SN, and PBC, potentially through pathways of information dissemination."  Regarding Pages 26; Lines 616-618. Clean version:  Original: "The analytical results further demonstrate that SNW exerts a significant positive effect on IBA (t=3.132, p=0.002). This relationship can be attributed to the dual mechanisms of information dissemination..."  Revision: We explicitly separated the empirical correlation from the theoretical interpretation by using phrasing such as "interpreting through the lens of..."  Revised Text: "The analytical results further demonstrate that SNW shows a significant positive correlation with IBA ($t=3.132,p=0.002$). Interpreting this through the lens of SNET, we suggest this relationship may be driven by the dual mechanisms of information dissemination..."  4. To strictly define the boundaries of our empirical claims and ensure readers approach the findings with appropriate caution, we have introduced a comprehensive new section, Section 6.3 Limitations. In this section, we explicitly address the constraints imposed by the research design. First, we clarify that the cross-sectional nature of the data precludes the determination of definitive causality; while our model is theoretically grounded, we openly acknowledge that the empirical results indicate statistical associations rather than proven directional effects. Furthermore, responding to your insightful comment regarding measurement validity, we have included a specific limitation concerning the scope of latent variables. We acknowledge that constructs such as "Emotional Network" are abstract theoretical concepts that are only imperfectly captured by the specific observed items in our questionnaire. By admitting that our interpretations are strictly bounded by the operational definitions used in this study, we prevent the generalization of findings beyond what the specific data supports.  The revised Section 6.3 is as follows:  Section 6.3 Limitations  Several limitations of this study should be acknowledged. First, the generalizability of the findings may be constrained by the relatively small sample drawn from a single coffee-producing region, which limits the applicability of the results to agricultural communities with distinct socioeconomic or geographical characteristics. Second, the measurement of emotional and suggestive networks relies on self-reported data, which could be influenced by social desirability bias, and the cross-sectional design precludes the establishment of causal relationships between network mechanisms and behavioral intentions. Third, while relational embeddedness is examined, structural properties of the networks are not accounted for, nor are potential moderating effects of individual differences across stakeholder types. Addressing these limitations in future research would strengthen the theoretical and practical contributions of this line of inquiry. ( Pages 35-36; Lines897-908. Clean version) |
| B. REQUEST FOR ADDITIONAL DETAILS  Future researchers may wish to reproduce the same results, on the same dataset, to confirm the agreement in methodology, before they apply a modification to the methods, investigate additional hypotheses, or replicate the study in another country, or countries, to investigate regional differences in the examined aspects. Therefore adding some technical details and clarifications would be highly desirable, and also required, in accordance with PLOS Publication Criterion no. 3: “Methods and reagents must be described in sufficient detail for another researcher to reproduce the experiments described.” Below is a list of items that would ideally be added to the main text or to the appendix, or otherwise (if not feasible) explained here. | |  |
| **Mathematical formulas** and other details allowing full replication. They do not have to be very formal, but they need to describe all procedures completely. Preferably to a Reader unfamiliar with Structural Equation Modelling, or one not in possession of the cited software. There are, for example, multiple ways to approach the problem of coefficient estimation, specific random seeds may be used in bootstrapping, etc., so obtaining the exact same coefficient values as the Authors did is not currently possible.  Selected specific questions (not an exhaustive list):   a. What is the exact objective function being optimized in process of estimation of the coefficients of the model? b. What is the nature of the estimated coefficients? Presumably the model is represented by a set of linear equations? It would be good to have them written out explicitly, in addition to the flowchart with arrows. c. How exactly is the significance of the indirect paths assessed? How is the T-statistic calculated for the indirect paths, what is the (\|O/STDEV\|) in Table 4?  2. (optional) It would be desirable to add the **intermediate dataset** to the raw data uploaded online, that is, the outcome of the iterative model estimation, with the numbers used to calculate the T statistics. In other words, everything required to follow all calculations exactly, from original raw data to final estimates. 3. **Original questions** (in original spelling, in the language in which they were asked, e.g. if there was a typo, it should be retained exactly as it was) should be included in the dataset or the appendix, just like the demographic questions are, in case of some future doubts about translation. 4. What is the (Likert) **scale of the answers**, how was it formulated? Does 1 mean Strongly Agree and 5 Strongly Disagree, in every question? Was there any transformation, truncation, any modification to originally recorded values at all? Were any values missing, if so, how many, which, how were they handled? 5. How are answers to individual questions in the questionnaire averaged to obtain values of the **latent variables**? Arithmetic mean, median? | | We sincerely appreciate this essential suggestion regarding the reproducibility and mathematical rigor of our work. We acknowledge that the previous version relied too heavily on software references (SmartPLS) without providing the underlying mathematical formulation, which limited the ability of readers—especially those unfamiliar with specific SEM software—to replicate the results. To address this, we have added a new dedicated section, Section 3.3 Statistical Analysis Details, where we have explicitly documented the mathematical procedures and estimation settings as follows:  Firstly, regarding the exact objective function, we have clarified in Section 3.3.2 that unlike covariance-based SEM, the Partial Least Squares (PLS-SEM) algorithm employed in this study uses an iterative approach to maximize the explained variance ($R^{2}$) of the endogenous latent variables. The specific objective is to minimize the residual variances ($\zeta$) in the structural equations, ensuring the model focuses on predictive power rather than global covariance fit. ( Pages 15; Lines 358-377. Clean version)  Secondly, to specify the nature of the estimated coefficients and the linear equations, we have revised Section 3.3.1 to include the explicit mathematical model. We clarified the Measurement Model (outer model) by defining the relationship between indicators and latent variables as $x_{jh}=\lambda_{jh}\xi_{j}+\varepsilon_{jh}$. Furthermore, the Structural Model (inner model) is now explicitly presented as a system of linear equations; for instance, the core equation for Perception of Outcome (PO) is defined as ( Pages 15; Lines 378–383. Clean version)  $PO=\beta_{1}IBA+\beta_{2}SN+\beta_{3}PBC+\beta_{4}ENW+\beta_{5}SNW+\zeta_{PO}$  This ensures that the estimated coefficients ($\beta$ and $\gamma$) are mathematically defined beyond simple flowcharts.  Thirdly, concerning the significance of indirect paths, we have detailed the bootstrapping and T-statistic calculation in Section 3.3.3. We specified that we employed the Bias-Corrected and Accelerated (BCa) Bootstrapping method with 5,000 subsamples. The T-statistics reported in our tables were calculated using the formula $t=O/STDEV$, where $O$ is the original sample estimate and $STDEV$ represents the standard deviation of the bootstrap distribution (the standard error). For indirect paths, the significance was assessed by calculating the product of the path coefficients for each bootstrap subsample, with the T-statistic derived from the standard deviation of these products. ( Pages 15-16; Lines 384-395. Clean version)  We believe these additions provide all the necessary technical parameters and mathematical formulations to allow future researchers to fully replicate our study using the raw dataset.  2. We fully embrace this suggestion to maximize the transparency and reproducibility of our study. We acknowledge that providing raw data alone is insufficient for verifying the specific calculation of T-statistics derived from iterative procedures. To address this, we have prepared and uploaded two additional supplementary files to our data repository to ensure full replicability:  (1) We have exported the comprehensive SmartPLS Report (in Excel format) and added it to the dataset. This file contains the detailed bootstrapping iteration matrices and the specific subsample estimates used to derive the standard errors and T-statistics reported in the manuscript.  (2) We have created a dedicated Operational Protocol (Word document) that documents the exact operational procedures used in SmartPLS. This document details every parameter setting and provides a step-by-step walkthrough of the software execution to allow any researcher to reproduce our final estimates exactly from the original raw data.  3. We sincerely appreciate this suggestion to ensure the transparency and reproducibility of our study. As requested, we have included the full list of original questions in their source language (Chinese) in Dataset B. These items have been retained exactly as they were presented to the respondents (including original spelling) to facilitate any future verification of translation accuracy.  4. We would like to thank you for your practical and rigorous questions regarding our measurement scales and data processing procedures. Ensuring the transparency of data collection and the integrity of the original values is vital for the reproducibility of this study. We have explicitly clarified the measurement scales and data cleaning procedures in the revised Section 4.2.1. ( Pages 18 Lines 464-469. Clean version)   （1） We specified that a standard 5-point Likert scale was used, where 1 represented "Strongly Disagree" and 5 represented "Strongly Agree". （2） We confirmed that no transformation, truncation, or modification was applied to the originally recorded values (raw integers were used). （3） We stated that the final dataset ($N=137$) contained zero missing values, as incomplete responses were systematically excluded during the initial data screening phase.   5. We would like to express our gratitude for your technical inquiry regarding the calculation of latent variable scores. This is a critical methodological detail that ensures the mathematical rigor of our Structural Equation Modeling (SEM) analysis.  To address your question about whether individual questionnaire items were averaged via arithmetic mean or median, we have added a new section, Section 3.4.4 Calculation of Latent Variable Scores, to the revised manuscript. This section provides a detailed mathematical explanation of the scoring process used in our study. ( Pages 16 Lines 396–405. Clean version)  We clarified that within the PLS-SEM framework, latent variable scores were not calculated using simple arithmetic means or medians. Instead, they were derived as weighted linear combinations of their respective indicators:   - $\xi_{j}=\sum w_{jh}x_{jh}$   Where $w_{jh}$ represents the outer weights determined by the PLS algorithm. This explanation corrects the potential misconception that unweighted averages were used and aligns our description with standard PLS-SEM methodology.  The revised Section 3.3 Statistical analysis details is as follows:  3.3 Statistical analysis details  3.3.1 Model specification  The Partial Least Squares Structural Equation Modeling (PLS-SEM) employed in this study consists of two sub-models: the measurement model (outer model) and the structural model (inner model). Since all latent variables (IBA, SN, PBC, PO, ENW, SNW) are conceptualized as reflective constructs, the Measurement Model is expressed by the linear Equation (1)  $\text{x}_{\text{j}\text{h}}\text{=}\text{λ}_{\text{j}\text{h}}\text{ξ}_{\text{j}}\text{+}\text{ε}_{\text{j}\text{h}}$ (1)  Where $\text{x}_{\text{j}\text{h}}$ represents the $\text{h}$-th indicator of the $\text{j}$-th latent variable $\text{ξ}_{\text{j}}$, $\text{λ}_{\text{j}\text{h}}$ is the outer loading (correlation between the indicator and the latent variable), and $\text{ε}_{\text{j}\text{h}}$ is the measurement error.  The Structural Model, representing the hypothesized relationships in Figure 2, is defined by the following system of linear equations (2):  $\text{ξ}_{\text{Endogenous}}\text{=∑}\text{β}_{\text{ji}}\text{ξ}_{\text{i}}\text{+}\text{ζ}_{\text{j}}$ (2)  Specifically, for the core outcome variable (PO), the Equation (3) is:  $\text{PO}\text{=}\text{β}_{\text{1}}\text{IBA}\text{+}\text{β}_{\text{2}}\text{SN}\text{+}\text{β}_{\text{3}}\text{PBC}\text{+}\text{β}_{\text{4}}\text{ENW}\text{+}\text{β}_{\text{5}}\text{SNW}\text{+}\text{ζ}_{\text{PO}}$ (3)  Similarly, the mediating variables are modeled as ( equations (4)-(6)):  $\text{IBA}\text{=}\text{γ}_{\text{1}}\text{ENW}\text{+}\text{γ}_{\text{2}}\text{SNW}\text{+}\text{ζ}_{\text{IBA}}$ (4)  $\text{SN}\text{=}\text{γ}_{\text{3}}\text{ENW}\text{+}\text{γ}_{\text{4}}\text{SNW}\text{+}\text{ζ}_{\text{SN}}$ (5)  $\text{PBC}\text{=}\text{γ}_{\text{5}}\text{ENW}\text{+}\text{γ}_{\text{6}}\text{SNW}\text{+}\text{ζ}_{\text{PBC}}$ (6)  Where $\text{β}$ and $\text{γ}$ represent the path coefficients to be estimated, and $\text{ζ}$ represents the residual variance (structural error).  3.3.2 Estimation and objective function  Unlike covariance-based SEM, PLS-SEM does not optimize a global fit function (such as minimizing the difference between observed and estimated covariance matrices). Instead, it employs an iterative algorithm to maximize the explained variance ($\text{R}^{\text{2}}$) of the endogenous latent variables. The objective function is to minimize the residual variances ($\text{ζ}$) in the structural equations.  3.3.3 Bootstrapping and significance testing  To assess the statistical significance of path coefficients and indirect effects, this study employed the Bias-Corrected and Accelerated (BCa) Bootstrapping method with 5,000 subsamples under a two-tailed testing framework ($\text{α}\text{=0.05}$). The bootstrapping algorithm achieved convergence after three iterations, ensuring the computational stability of the standard error estimates. Specifically, the T-statistics reported in Tables 3 and 4 were calculated as the ratio of the original sample estimate ($\text{O}$) to the standard error ($\text{STDEV}$) derived from the bootstrap distribution ($\text{t}\text{=}\text{O}\text{/}\text{STDEV}$), where $\text{STDEV}$ represents the standard deviation of the 5,000 bootstrap estimates. Furthermore, the significance of the mediation path was assessed by calculating the product of coefficients for each bootstrap subsample. The mean of these products served as the point estimate, while their standard deviation across subsamples functioned as the standard error for the T-test.  3.3.4 Calculation of Latent Variable Scores  It is important to clarify that the values of the latent variables used in the structural model were not derived from simple arithmetic means or medians of the observed indicators. Instead, consistent with the PLS-SEM algorithm, latent variable scores were calculated as weighted linear combinations of their respective indicators (Equation (7)):  $\text{ξ}_{\text{j}}\text{=∑}\text{w}_{\text{j}\text{h}}\text{x}_{\text{j}\text{h}}$ (7)  Where $\text{w}_{\text{j}\text{h}}$ represents the outer weights determined by the PLS algorithm to maximize the explained variance, and $\text{x}_{\text{j}\text{h}}$ represents the standardized values of the observed indicators. This method accounts for the varying contribution of each indicator to the construct, offering higher precision than unweighted averaging. ( Pages 14-16 Lines 357-405. Clean version) |
| **Optional additions:**   1. It would be very desirable (but not absolutely compulsory) to add another column to the online dataset, specifying whether the answers from a participant were obtained **online or in-person**. Ideally, a code designated the interviewer would be added (so that one could distinguish which participants were interviewed by the same person). 2. If possible, another column could be added, reporting **relationships between participants**: was the participant interviewed together with another participant (if so, which)? Have multiple members of the same family, or housemates, been invited to the survey? It is conceivable that people who live together, or are close friends, partners, co-workers, etc., share certain attitudes and convictions, or that they fill out the questionnaire together, in which case their answers may be influenced by the concern about the opinion of the other participant about their answers. Such additional information could therefore provide valuable insights in the future, plus, would allow to assess the independence of the sampled data, even if the information is only available for a subset of participants. But I understand that participants may not wish to provide such information, there may be additional personal data protection ramifications, etc., so please consider it more a suggestion that request. 3. Was the survey anonymous, were any participants personally known to the interviewers?   Were any participants (if so, which) already actively involved in the comanagement? Again, this is optional. | | We would like to express our sincere appreciation for your thoughtful and constructive suggestions regarding the transparency of our dataset and the context of our survey implementation. Your insights regarding data independence and potential respondent bias are highly valuable for enhancing the rigor and future utility of our research.  To address these optional yet important points, we have carefully evaluated our data records and updated the manuscript and dataset where possible, while maintaining our commitment to participant privacy.  1. We appreciate this valuable suggestion to enhance data transparency and have updated the online dataset accordingly. Specifically, we have added two new columns at the beginning of Dataset B: the first column specifies whether the data was obtained online or in-person, and the second column provides the corresponding interviewer code to identify the data collector.  Regarding the specific roles associated with these codes, two interviewers were involved in this study. Interviewer 1 was primarily responsible for conducting the in-person surveys, while Interviewer 2 was primarily responsible for the online surveys distributed via the Wenjuanxing platform.  2. We fully acknowledge the theoretical importance of assessing data independence and potential peer influence among participants. However, as the reviewer kindly anticipated, our data collection was conducted under a strict anonymity protocol to encourage honest responses and protect participant privacy. Consequently, we did not record any information regarding personal relationships between participants (such as family ties or friendships) or group participation dynamics. We sincerely apologize that, due to these privacy constraints, we are unable to include this additional column in the dataset.  3. We sincerely appreciate the reviewer raising this important point regarding sample objectivity and potential bias. We would like to respectfully clarify the relationship between the interviewers and the participants:  To ensure the impartiality of the data, the survey was conducted with strict independence. Specifically, Interviewer 1 adhered to a protocol of conducting in-person interviews exclusively with strangers (individuals personally unknown to the interviewer). Meanwhile, Interviewer 2 managed the online survey, which was designed to be completely anonymous. Therefore, we can confirm that no participants were personally known to the interviewers.  Regarding your thoughtful inquiry about whether specific participants were already actively involved in co-management: While we recognize the value of this background information, the strict anonymity measures mentioned above prevent us from identifying individual participants or tracking their specific prior involvement. We sincerely regret that we cannot provide this specific detail, but we hope the reviewer understands that this limitation is a result of our commitment to protecting participant privacy.  In summary, we have incorporated your suggestions to the maximum extent permitted by our original research design and ethical protocols. We believe these additional details regarding interviewer roles and data collection modes significantly improve the transparency of our empirical work. We hope these clarifications and the updated dataset meet your expectations and further strengthen the validity of our findings. |
| OPTIONAL SUGGESTIONS AND COMMENTS    Overall, the text is well-written; even if it could benefit from a little less jargon in some places, especially the Introduction.  It lack a discussion of limitations of methodology, and statistical analysis in particular. | |  |
| A short paragraph on future work (what else could be done on this subject, logical next research questions to investigate, alternative approaches or models to pursue, perhaps with other data or resources) would help put the study into perspective. | | We sincerely thank the reviewer for the insightful suggestion regarding the inclusion of a future work section to contextualize the study further. In response, we have strengthened the concluding discussion by adding a dedicated subsection titled 6.3 Limitations at the end of the paper.  This section acknowledges several constraints of the current study, which naturally point toward promising directions for future research. Specifically, we note that:  The generalizability of findings may be constrained by the sample drawn from a single region, suggesting the value of cross-regional or cross-cultural comparisons in future work.  The reliance on self-reported cross-sectional data limits causal inference, indicating that longitudinal or experimental designs could be meaningful next steps.  The focus on relational embeddedness, without accounting for structural network properties or individual differences across stakeholder types, invites future studies to integrate multidimensional network measures or moderating variables.  By framing these limitations explicitly, we aim to not only enhance the transparency and scholarly rigor of the manuscript but also outline logical extensions of this research—aligning closely with your constructive recommendation to “help put the study into perspective.”  The revised Section 6.3 is as follows:  6.3 Limitations  Several limitations of this study should be acknowledged. First, the generalizability of the findings may be constrained by the relatively small sample drawn from a single coffee-producing region, which limits the applicability of the results to agricultural communities with distinct socioeconomic or geographical characteristics. Second, the measurement of emotional and suggestive networks relies on self-reported data, which could be influenced by social desirability bias, and the cross-sectional design precludes the establishment of causal relationships between network mechanisms and behavioral intentions. Third, while relational embeddedness is examined, structural properties of the networks are not accounted for, nor are potential moderating effects of individual differences across stakeholder types. Addressing these limitations in future research would strengthen the theoretical and practical contributions of this line of inquiry. (Page 35-36; Lines 897-908. Clean version)  Thank you again for this valuable suggestion, which has helped us improve the completeness and forward-looking tone of the conclusion. |
| The ending is slightly abrupt. A distinctive, impactful **closing paragraph** referring back to the main objectives and accomplishments would improve overall impression. | | Thank you for your valuable suggestion regarding the need for a more impactful and conclusive ending. In response, we have substantially revised and expanded the Conclusion section to provide a clearer synthesis of the study's key objectives and contributions.  Specifically, we have added a dedicated closing paragraph that succinctly recaps the core findings in relation to the original research aims. This paragraph highlights how the integration of the Theory of Planned Behavior and Social Network Embeddedness Theory offers a unified framework for understanding multi-stakeholder environmental behavior, emphasizing that social networks not only complement cognitive factors but actively reshape them through affective and informational pathways. The passage is as follows:  From a practical perspective, the results highlight that intrinsic motivation and self-efficacy outweigh normative pressure in driving ecological co-management intentions within agricultural settings. This underscores the need to prioritize attitude change and capability building alongside interventions based on social norms. By bridging the TPB and SNET, a unifying framework is provided for explaining multi-stakeholder environmental behavior, demonstrating that social networks do not merely supplement cognitive factors but actively reshape them through affective and informational mechanisms. (Page 33-34; Lines 834-840. Clean version)  We believe this strengthened conclusion enhances the overall coherence of the paper and leaves the reader with a clearer understanding of the study's theoretical and practical significance. |
| The legends of the demographic data pie charts (Figure 2) are not entirely legible, due to the use of similar colors and no alternative way to connect the labels with the parts of the chart, other than by reading the main text. Perhaps adding a background line pattern would be helpful, or positioning labels next to the pieces that they correspond to. Such improved accessibility may be particularly appreciated by color-blind (or visuallyimpaired in other ways) Readers. | We strictly followed this thoughtful suggestion and sincerely thank the reviewer for highlighting the importance of accessibility for all readers, including those with color vision deficiencies.  To address this, we have completely redesigned Figure 2 to eliminate reliance on color distinction. Specifically, instead of using a separate legend with similar colors, we have positioned the labels directly next to their corresponding chart segments (connected by leader lines where necessary). This modification ensures that the demographic data is now clearly legible and unambiguous, regardless of the reader's ability to distinguish colors. (Page 21; Lines 530-531. Clean version)    Fig. 2. Demographic results of the survey respondents | |
| I found Figure 3 (flowchart illustration of the model) slightly confusing at first; it cleared up after understanding the model from reading the text. I understand it is difficult to graphically distinguish Hypothesis 2 from Hypothesis 4. So this is just an optional suggestion: I would consider repositioning the boxes so that all arrows point from left to right. That would focus the Reader’s attention immediately on the meaning rather than direction of the arrows. It would also make the absence of cycles immediately clear.  Whatever the Authors’ decision with respect to Figure 3, I would strongly advise to keep Figure 4 consistent with Fig. 3, that is, for it to have the same layout of latent variables. Again, I would personally prefer to see the ENW and SNW on the left and IBA, SN, PBC in the middle, but I am not particular about this. | | We sincerely appreciate your insightful suggestion regarding the visual presentation of our model. In accordance with your advice, we have redesigned Figure 3 so that all arrows now point from left to right. This adjustment clearly illustrates the absence of cycles and focuses attention on the causal relationships. Furthermore, we have updated Figure 4 to ensure it shares the exact same layout as Figure 3. Specifically, we have positioned ENW and SNW on the left, with IBA, SN, and PBC in the middle. We believe these changes have made the illustrations much clearer and easier to follow. Additionally, in the revised manuscript, the redesigned original Figure 3 is renumbered as Figure 1, and the updated original Figure 4 is renumbered as Figure 3.    Fig.1. The conceptual model of this paper.  (Page 14; Lines 355-356. Clean version)    Fig. 3. Paths of structural equation model  (Page 24; Lines 576-577. Clean version) |
| The **order of latent variables** should be the same in every table and figure. For example, in Table 2, it differs for no apparent reason.  **Table 3** could also have a slightly different order of rows, for example, corresponding to the order of paths in Fig. 4, starting with top left. But if there is another pattern I did not notice at first, that is fine too.  **Typos:**   - Line 514: “Furthermore, Consistent” – capitalization error - Line 384: SNWT instead of SNET (I presume) - Online dataset: IBA questions labelled as IA | | We appreciate your keen attention to detail regarding the consistency of the data presentation. We agree that maintaining a uniform order for latent variables across all tables and figures is essential for clarity.  We have carefully revised the manuscript to standardize the order of variables throughout. Specifically, Table 2 has been updated to match the sequence used in the figures. Furthermore, we have reorganized the rows in Table 3 to correspond with the logical flow of paths shown in Figure 3 (starting from the top left), as suggested. These changes ensure a consistent reading experience across the entire paper. |
|  | | Dear Reviewer,  We would like to thank you again for the time you spent reading our manuscript and providing the helpful comments above. As you can see, we have made every effort to address them. We hope that you will be satisfied with our responses to these comments. Thank you for your work. |

**References**

8.	Granovetter, M., *Economic Action and Social Structure: The Problem of Embeddedness.* American Journal of Sociology, 1985. **91**(3): p. 481-510.

13.	Wang, Y., J. Zhao, and J. Pan, *The investigation of green purchasing behavior in China: A conceptual model based on the theory of planned behavior and self-determination theory.* Journal of Retailing and Consumer Services, 2024. **77**: p. 103667.

14.	Zhu, D., et al., *The influence of psychological cognition and policy environment on the basin residents' behavior of ecological compensation under the background of carbon neutrality: A case study in upper Yellow River basin, China.* Ecological Indicators, 2023. **148**: p. 110031.

15.	Bortne, Ø., et al., *Self-efficacy or perceived behavioral control: which influences bank-switching intention?* Journal of Marketing Analytics, 2025.

16.	Villanueva-Flores, M., et al., *Exploring the mediation role of perceived behavioural control and subjective norms in the relationship between psychological capital and entrepreneurial intention of university students.* The International Journal of Management Education, 2023. **21**(3): p. 100865.

17.	Lou, S., X. Zhang, and D. Zhang, *Research on the influence mechanism of residents’ waste sorting behavior: based on extended theory of planned behavior.* Environment, Development and Sustainability, 2024.

18.	Lalani, B., et al., *Smallholder farmers' motivations for using Conservation Agriculture and the roles of yield, labour and soil fertility in decision making.* Agricultural Systems, 2016. **146**: p. 80-90.

19.	Huang, Y., et al., *Predicting citizens’ participatory behavior in urban green space governance: Application of the extended theory of planned behavior.* Urban Forestry & Urban Greening, 2021. **61**: p. 127110.

20.	Yu, X., P. Wang, and Y. Li, *Governance activities, villagers' awareness, characteristics, and willingness in rural environmental participation: evidence from Fujian, China.* Environment, Development and Sustainability, 2024. **26**(11): p. 29075-29097.

21.	Zhang, K., et al., *Revealing urban residents’ intention to pay for the greening of farmland in the urban fringe by extending the theory of planned behavior: Insights from payment for ecosystem services.* Land Use Policy, 2024. **141**: p. 107127.

22.	Nell, P.C. and U. Andersson, *The complexity of the business network context and its effect on subsidiary relational (over-) embeddedness.* International Business Review, 2012. **21**(6): p. 1087-1098.

23.	Frankowska, M. and K. Cheba, *The relational embeddedness as the differentiator of the cluster supply chain collaboration – a multidimensional comparative analysis.* Competitiveness Review, 2021. **32**(1): p. 59-84.

24.	Swierczek, A., *Manufacturer structural embeddedness and the network rent: the intervening role of relational embeddedness in the triadic supply chains.* Supply Chain Management: An International Journal, 2019. **24**(3): p. 334-354.

27.	Li, J., et al., *The impact of social network embeddedness, TPB, and danwei system on residents' participation intention in old neighborhood regeneration.* Scientific Reports, 2024. **14**(1): p. 18920.

28.	Ahmed, H., L. Ekman, and N. Lind, *Planned behavior, social networks, and perceived risks: Understanding farmers' behavior toward precision dairy technologies.* Journal of Dairy Science, 2024. **107**(5): p. 2968-2982.

30.	La Barbera, F. and I. Ajzen, *Understanding Support for European Integration Across Generations: A Study Guided by the Theory of Planned Behavior.* Europe’s Journal of Psychology, 2020. **16**(3).

31.	Zhong, Y., et al., *Keeping things cordial: Competition, cooperation and coopetition in venture capital syndicates.* Journal of Business Research, 2025. **201**: p. 115638.

33.	Liao, C.-H., *Exploring social media determinants in fostering pro-environmental behavior: insights from social impact theory and the theory of planned behavior.* Frontiers in Psychology, 2024. **Volume 15 - 2024**.

34.	Holdsworth, S., et al., *The assessment of graduate sustainability attributes in the workplace: Potential advantages of using the theory of planned behaviour (TPB).* Journal of Cleaner Production, 2019. **238**: p. 117929.

35.	Wang, Z., et al., *Exploring local residents’ response to ecological resettlement policy: a TPB-based study in Zhalong National Nature Reserve.* World Development Sustainability, 2025. **7**: p. 100246.

36.	Yin, J. and S. Shi, *Social interaction and the formation of residents′ low-carbon consumption behaviors: An embeddedness perspective.* Resources, Conservation and Recycling, 2021. **164**: p. 105116.

37.	Czyżewski, B., et al., *Exploring intentions to convert into organic farming in small-scale agriculture: Social embeddedness in extended theory of planned behaviour framework.* Agricultural Systems, 2025. **225**: p. 104294.

38.	Malau, L.R.E., et al., *Why we waste: A meta-analysis of food waste behavior using the Theory of Planned Behavior (TPB).* Sustainable Futures, 2025. **10**: p. 101421.

39.	Dogantekin, A., C. Secilmis, and O.M. Karatepe, *Qualitative job insecurity, emotional exhaustion and their effects on hotel employees’ job embeddedness: The moderating role of perceived organizational support.* International Journal of Hospitality Management, 2022. **105**: p. 103270.

40.	Zhang, D., T. Yang, and S. Lou, *Combining Maslow's hierarchy of needs theory with the TPB: What influences waste separation behavior in Chinese residents: Evidence from Shanghai.* Sustainable Futures, 2025. **9**: p. 100660.

41.	Izquierdo-Yusta, A., M.P. Martínez–Ruiz, and H.H. Pérez–Villarreal, *Studying the impact of food values, subjective norm and brand love on behavioral loyalty.* Journal of Retailing and Consumer Services, 2022. **65**: p. 102885.

42.	Swami, D. and D. Parthasarathy, *Role of intrinsic motivation and government policies in adoption of sustainable agriculture practices by farmers in Maharashtra, India.* Farming System, 2024. **2**(3): p. 100100.

43.	Li, Q., Q. Zhai, and J. Wang, *The impact of information intervention on urban residents' willingness to sort domestic waste.* Journal of Environmental Management, 2024. **371**: p. 123201.

44.	Ushchev, P. and Y. Zenou, *Social norms in networks.* Journal of Economic Theory, 2020. **185**: p. 104969.

45.	Xu, Z., et al., *The influencing factors of residents’ willingness to pay in marine ecological restoration: The integration of the theory of planned behavior and social capital theory.* Marine Policy, 2024. **161**: p. 106031.

46.	Araújo, A., et al. *Bridging the Attitude–Behavior Gap in Sustainable Tourism: An Extended TPB Model of Green Hotel Purchase Intentions*. Tourism and Hospitality, 2025. **6**, 215 DOI: 10.3390/tourhosp6040215.

47.	Ataei, P., et al., *An analysis of farmers’ intention to use green pesticides: The application of the extended theory of planned behavior and health belief model.* Journal of Rural Studies, 2021. **81**: p. 374-384.

48.	Govindan, K., Y. Zhuang, and G. Chen, *Analysis of factors influencing residents' waste sorting behavior: A case study of Shanghai.* Journal of Cleaner Production, 2022. **349**: p. 131126.

49.	Sawaneh, I.A., L. Fan, and B. Sesay, *Investigating the influence of residents' attitudes, perceptions of risk, and subjective norms on their willingness to engage in flood prevention efforts in Freetown, Sierra Leone.* Nature-Based Solutions, 2024. **6**: p. 100143.

50.	Nguyen Thi Huyen, N., H. Duong Hong, and L. Hoang Thi, *Green practices: Building green image and green trust for green revisit intentions in the hospitality industry.* Journal of Open Innovation: Technology, Market, and Complexity, 2025. **11**(1): p. 100481.

55.	Helfaya, A. and P. Bui, *Pursuing a corporate sustainable identity: Green governance strategy, hybrid vehicle development, knowledge and sustainability performance.* Journal of Innovation & Knowledge, 2025. **10**(2): p. 100660.

56.	Bouman, T., et al., *Environmental values and identities at the personal and group level.* Current Opinion in Behavioral Sciences, 2021. **42**: p. 47-53.

57.	Gorris, P. and L. Koch, *Building trust in environmental co-management: Social embeddedness in a contested German biodiversity conservation governance process.* Environmental Science & Policy, 2024. **154**: p. 103695.

58.	Marín, A., et al., *Disaster impacts on co-management networks: longitudinal and comparative analysis of Chilean small-scale fisheries.* Frontiers in Marine Science, 2023. **Volume 10 - 2023**.

59.	Nguyen, G.N.T. and T. Sarker, *Sustainable coffee supply chain management: a case study in Buon Me Thuot City, Daklak, Vietnam.* International Journal of Corporate Social Responsibility, 2018. **3**(1): p. 1.

60.	Brosch, T., *Affect and emotions as drivers of climate change perception and action: a review.* Current Opinion in Behavioral Sciences, 2021. **42**: p. 15-21.

61.	Bago, B. and W. De Neys, *Advancing the specification of dual process models of higher cognition: a critical test of the hybrid model view.* Thinking & Reasoning, 2020. **26**(1): p. 1-30.

62.	Jugert, P., et al., *Collective efficacy increases pro-environmental intentions through increasing self-efficacy.* Journal of Environmental Psychology, 2016. **48**: p. 12-23.

63.	Bamberg, S. and G. Möser, *Twenty years after Hines, Hungerford, and Tomera: A new meta-analysis of psycho-social determinants of pro-environmental behaviour.* Journal of Environmental Psychology, 2007. **27**(1): p. 14-25.

66.	Emmanouil, C., et al., *Application of the Theory of Planned Behavior to predict waste source separation.* Science of The Total Environment, 2024. **956**: p. 177356.
